# Newly Generated Atractylon Derivatives in Processed Rhizomes of *Atractylodes macrocephala* Koidz

**DOI:** 10.3390/molecules25245904

**Published:** 2020-12-13

**Authors:** Chunmei Zhai, Jianping Zhao, Amar G. Chittiboyina, Yonghai Meng, Mei Wang, Ikhlas A. Khan

**Affiliations:** 1 School of Pharmacy, Heilongjiang University of Chinese Medicine, Harbin 150040, China; zhaicm163@163.com (C.Z.); myhdx163@163.com (Y.M.); 2 National Center for Natural Products Research, School of Pharmacy, University of Mississippi, Oxford, MS 38677, USA; jianping@olemiss.edu (J.Z.); amar@olemiss.edu (A.G.C.); 3 Natural Products Utilization Research Unit, Agricultural Research Service, Department of Agriculture, University of Mississippi, Oxford, MS 38677, USA; mei.wang@usda.gov; 4 Division of Pharmacognosy, Department of BioMolecular Sciences, School of Pharmacy, University of Mississippi, Oxford, MS 38677, USA

**Keywords:** *Atractylodes macrocephala*, wheat bran, processing, NMR, atractylon derivatives, isolation and identification

## Abstract

Thermally processed rhizomes of *Atractylodes macrocephala* (RAM) have a long history of use in traditional Chinese medicine (TCM) for treating various disorders, and have been an integral part of various traditional drugs and healthcare products. In TCM, herbal medicines are, in most cases, uniquely processed. Although it is thought that processing can alter the properties of herbal medicines so as to achieve desired functions, increase potency, and/or reduce side effects, the underlying chemical changes remain unclear for most thermally processed Chinese herbal medicines. In an attempt to shed some light on the scientific rationale behind the processes involved in traditional medicine, the RAM processed by stir-frying with wheat bran was investigated for the change of chemical composition. As a result, for the first time, five new chemical entities, along with ten known compounds, were isolated. Their chemical structures were determined by spectroscopic and spectrometric analyses. The possible synthetic pathway for the generation of such thermally-induced chemical entities was also proposed. Furthermore, biological activity evaluation showed that none of the compounds possessed cytotoxic effects against the tested mammalian cancer and noncancer cell lines. In addition, all compounds were ineffective at inhibiting the growth of the pathogenic microorganisms.

## 1. Introduction

The rhizome of *Atractylodes macrocephala* Koidz (RAM)—called Baizhu in traditional Chinese medicine (TCM)—is a well-known herbal medicine. It was documented in the earliest existing book on TCM—“Shen Nong’s Materia Medica” written during the Han Dynasty (A.D. 25-220). RAM has been traditionally used for the treatment of various disorders, such as loss of appetite, diarrhea, limb weakness, gastrointestinal dysfunction, immune dysfunction, diabetes, and some chronic inflammatory diseases [1]. It was reported that RAM is used in more than 835 TCM preparations, as well as an integral part of more than 4340 classic prescriptions for treating chronic diseases [1]. Indeed, RAM is considered a functional food, a tonic, and a constituent of various health products purported for promoting digestion, alleviating fatigue, improving sleeping, enhancing immunity, and treating alimentary anemia [2]. RAM is traditionally used in its processed form which is commonly achieved by stir-frying raw RAM with wheat bran [3,4]. This type of processing technique, known as Pao-Zhi in TCM, has been widely used for the preparation of Chinese Materia Medica [5] with a long history. It is believed that after processing, property and function of remedies can be changed, medical potency can be increased, and/or toxicity and side effect can be reduced. The practice of processing RAM can date back to the Tang dynasty (A.D. 618–907) [6], and the earliest processing protocol for RAM by stir-frying with wheat bran was recorded in the famous Sheng Ji Zong Lu, the TCM prescription book written in A.D. 1117. This processing method is also currently documented in the Chinese Pharmacopeia [7]. Although processed RAM is commonly used as an ingredient of various Chinese medicines and health products, so far, the chemical compositional change as a result of processing has not been clearly delineated.

Extensive phytochemical and pharmacological studies have been conducted on raw RAM, revealing the presence of volatile organics, sesquiterpenoids, triterpenoids, polyacetylenes, coumarins and phenylpropanoids, flavonoids and their glycosides, steroids, benzoquinones, and polysaccharides [1]. Among them, sesquiterpene lactones (i.e., atractylenolids I, II, and III) are considered to be the characteristic and bioactive constituents of RAM [8,9]. Efforts have been previously undertaken to investigate the changes occurring in the chemical components of RAM before and after processing by utilizing HPLC, GC/MS, and other analytical techniques. Substantial changes in the concentration of constituents, including those in essential oil, as well as the eudesmane-type sesquiterpenoids (such as atractylon, atractylnolides I, II, and III, etc.), were observed [3,10,11,12,13]. These changes can be explained as the result of evaporation, conversion, and/or degradation by heating, due to their physicochemical properties such as volatility and instability [13,14]. Nevertheless, in continuation of the quest to probe the overall integrity of botanical preparations by using NMR-based metabolomics to investigate the change of chemical composition which occurred during processing, we observed that some new characteristic signals appeared in the NMR spectra of the processed RAM, which suggested that some unknown compounds could be generated during processing. Herein, the discovery of five new compounds isolated from the RAM processed by stir-frying with wheat bran is reported. Their chemical structures were determined by both spectroscopic and spectrometric analyses. In addition, a plausible mechanism for the generation of these new compounds was proposed. Furthermore, their cytotoxic, antimicrobial, and antileishmanial activities were evaluated.

## 2. Results and Discussion

### 2.1. Structure Elucidation

A previous study using a NMR-based metabolomics method to investigate the effects of processing on the Chinese herbal medicine *Flos Lonicerae* revealed that the NMR approach can provide not only a holistic view on the change of chemical composition during processing, but also the information on identity of individual components [5]. In the present study, with the aid of information obtained from the NMR-based metabolomic analyses of RAM before and after processing, we could narrow the scope of isolation targets to the hexane partition fraction and its sub-fractions based on new signals of interest observed in their NMR spectra.

**Figure 1:**
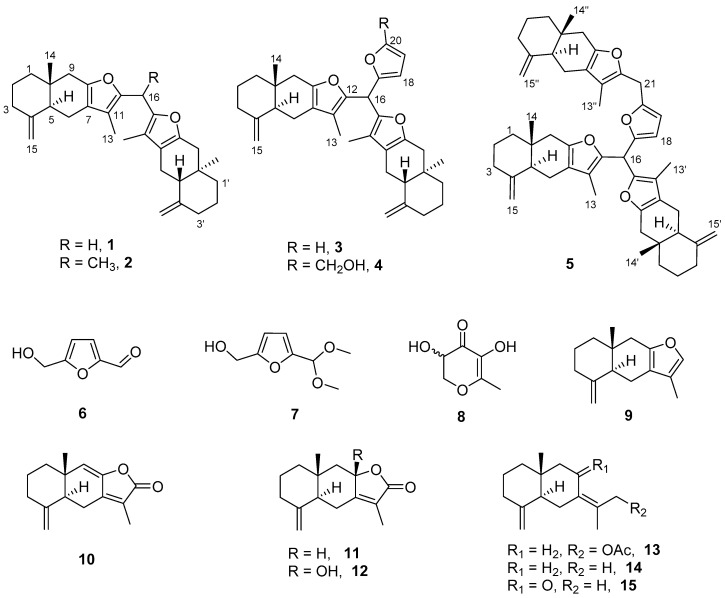
Structures of compounds **1**–**15**.

Compound **1** was obtained as a white amorphous powder. Its molecular formula was established as C_31_H_40_O_2_ based on its high-resolution atmospheric pressure chemical ionization mass data (HR-APCI-MS, *m/z* = 445.3134 [M + H]^+^). From its ^13^C-NMR spectrum, only 16 resonance signals were observed, suggesting **1** might contain two symmetrical sub-structures with 15 carbons for each structure. Referring to the multiplicity-edited HSQC spectrum, the signals in the ^13^C spectrum were assigned to two methyls, six methylenes, one exocyclic methylidene, one aliphatic methine, one aliphatic quaternary, and five olefinic carbons (Table 1). The ^1^H-NMR spectrum showed signals of methyl groups at *δ*_H_ 1.87 (s) and 0.76 (s), exocyclic methylidene at *δ*_H_ 4.68 and 4.84, aliphatic methine at *δ*_H_ 2.10 (m), five methylenes in the range of *δ*_H_ 1.40–2.50, and one methylene at *δ*_H_ 3.82 (s). The integral value of the peak of methylene at *δ*_H_ 3.82 was found to be only 1/3 of the value for the singlet peak of methyl at *δ*_H_ 0.76 or 1.87, indicating that this methylene is the group bridging the two symmetrical moieties together with each moiety containing 15 carbons (Figure 1). The HMBC spectrum of **1** reveals the cross-peaks of the methyl signal at *δ*_H_ 0.76 (H-14,14′) with the carbon signals at *δ*_C_ 42.0 (C-1,1′), *δ*_C_ 45.7 (C-5,5′), *δ*_C_ 39.2 (C-9,9′) and *δ*_C_ 36.7 (C-10,10′). Additionally, the cross-peaks of the methyl signal at *δ*_H_ 1.87 (H-13,13′) with the carbon signals at *δ*_C_ 116.6 (C-7,7′), *δ*_C_ 114.3 (C-11,11′), and *δ*_C_ 145.0 (C-12,12′), and the cross-peaks of the exocyclic methylidene at *δ*_H_ 4.68 and 4.84 (H-15,15′) with the carbon signals at *δ*_C_ 37.3 (C-3,3′), *δ*_C_ 150.0 (C-4,4′), and *δ*_C_ 45.7 (C-5,5′) were observed in the HMBC spectrum. These observations suggested that the 15-carbon moiety had a furanoeudesmane carbon skeleton in the structure as in atractylon, which is a known furanosesquiterpene isolated from RAM in significant quantity [15]. The analysis of the ^1^H-^1^H COSY spectrum further confirmed the assignment. The nuclear Overhauser effect (NOE) interactions between H-14 (14′) and H-2β (2′β)/H-6β (6′β), but no NOE interaction between H-14 (14′) and H-5 (5′), were observed in the NOESY spectrum, indicating that the *trans*-fused A/B ring junction and α,β-orientation of H-5 (5′) and Me-14 (14′) in the furanoeudesmane skeleton was the same as atractylon. Observations of HMBC correlations of the methylene signal at *δ*_H_ 3.82 (H-16) to C-11 (11′) at *δ*_C_ 114.3 and C-12 (12′) at *δ*_C_ 145.0 in the HMBC spectrum indicated that the two symmetrical moieties were linked together through the methylene group attaching to C-11 and C-11′. Accordingly, **1** was identified to be bis(3,8α-dimethyl-5-methylene-4,4α,5,6,7,8,8α,9-octahydronaphtho-[2,3-β]-furan-2-yl)methane, trivially named methylene-biatractylon.

Compound **2** was obtained as a white amorphous powder. As determined from the [M + H]^+^ peak at *m/z* = 459.3267 in HR-ACPI-MS, **2** has a molecular formula of C_32_H_42_O_2_ with 12 degrees of unsaturation (DOU). Compound **2** has the same number of DOU as compound **1**, but has an additional CH_2_ in molecular formula. The ^1^H NMR and HSQC spectra of **2** exhibit similar signal patterns as those of **1**, respectively, i.e., the signals of methyl groups at *δ*_H_ 1.82/1.76 (s) and 0.75/0.77 (s), exocyclic methylidene at *δ*_H_ 4.68 and 4.84, aliphatic methine at *δ*_H_ 2.10 (m), six methylenes in the range of *δ*_H_ 1.40–2.50. A significant difference lies in that one methylene at *δ*_H_ 3.82 (s) was observed for **1**, but one aliphatic methine at *δ*_H_ 4.17 (q) and one methyl at *δ*_H_ 1.57 (d) were observed for **2** (Table 1). The ^13^C-NMR spectrum of **2** also displays a similar signal pattern as that of **1**. A significant difference between **2** and **1** is the presence of one methine carbon at *δ*_C_ 30.1 and one methyl carbon at *δ*_C_ 18.1 in **2**, corresponding, respectively, to the proton signals at *δ*_H_ 4.17 and *δ*_H_ 1.57. The analyses of the COSY and HSQC spectra confirmed the presence of an ethylidene unit in **2**, instead of the presence of a methylene in **1**. Comparing **2** to **1**, another difference in their ^13^C spectra is that slight splitting of carbon signals for the pair groups of C-1/1′, C-5/5′, C-9/9′, C-11/11′, C-13/13′, and C-14/14′ were observed for **2** but not for **1** (Table 1). Analyses of the HMBC and COSY spectra of **2** revealed that these pair of signals were attributed to the two atractylon moieties presented in **2**. Furthermore, the *trans*-fused A/B ring junction in the two atractylon moieties was confirmed by the NOE observations from the NOESY spectrum. The HMBC correlations of the methyl protons (Me-17) in the ethylidene unit at *δ*_H_ 1.75 with the carbons C-16 at *δ*_C_ 30.1, C-11 (11′) at *δ*_C_ 112.9/113.0, and C-12 (12′) at *δ*_C_ 149.1, and the methine proton (H-16) in the ethylidene unit at *δ*_H_ 4.17 with the carbons C-17 at *δ*_C_ 18.1, C-11 (11′) at *δ*_C_ 112.9/113.0, and C-12 (12′) at *δ*_C_ 149.1 indicated that the two atractylon moieties were linked together through the methine in the ethylidene unit connecting at C-11 and C-11′. On the basis of the aforementioned evidence, the structure of **2** was established to be 2,2′-(ethane-1,1-diyl)-bis(3,8α-dimethyl-5-methylene-4,4α,5,6,7,8,8α,9-octahydronaphtho-[2,3-β]-furan), trivially named ethylidene-biatractylon.

Compound **3** was isolated as a white amorphous powder. Its molecular formula was determined as C_35_H_42_O_3_ based on the HR-ACPI-MS data for the [M + H]^+^ peak at *m/z* = 511.3221, accounting for 15 degrees of unsaturation. Both the ^1^H and ^13^C signals of **3** in the upfield NMR chemical shift range showed close similarities to those of **1** and **2**, however, differences were observed in the downfield range. Four additional carbon signals at *δ*_C_ 153.1, 141.4, 110.2, and 107.2 in the ^13^C spectrum, and three additional proton signals at *δ*_H_ 7.35 (d), 6.30 (dd), and 6.07 (d) in the ^1^H spectrum were observed for **3**, as compared to **1** or **2**. These signals were assigned to a furan ring (3 degrees of unsaturation) by the analyses of COSY and HMBC spectra. Apart from these differences, the other signals displayed almost the same features as in **1** or **2.** The analyses of the 2D NMR spectra confirmed that **3** possessed a similar biatractylon skeleton in its structure as was found in **1** and **2**. Clear HMBC correlations of the methine proton (CH-16, with proton and carbon signals at *δ*_H_ 5.47 (s) and *δ*_C_ 36.1, respectively) to C-17 at *δ*_C_ 153.1, C-18 at *δ*_C_ 107.2, C-12 at *δ*_C_ 144.7, and C-11 *δ*_C_ 115.2 were observed, indicating that the furan ring and the two atractylon moieties were linked together through this methine. Hence, the structure of **3** was determined to be 2,2′-(furan-2-yl-methylene)-bis(3,8α-dimethyl-5-methylene-4,4α,5,6,7,8,8α,9-octahydronaphtho-[2,3-β]-furan), trivially named furan-2-methanetriyl-biatractylon.

Compound **4** was obtained as a white amorphous powder. The [M + H]^+^ ion at *m/z* = 541.3299 in the HR-ACPI-MS spectrum revealed its molecular formula as C_36_H_44_O_4_, with the same 15 degrees of unsaturation but with one more carbon, two more protons, and one more oxygen when compared to **3**. The ^1^H-NMR spectrum of **4** showed almost the same features as that of **3**, except for having one more singlet signal at *δ*_H_ 4.55. The ^13^C-NMR spectrum of **4** also displayed close similarity to that of **3**, except for the addition of an oxygenated methylene signal at *δ*_C_ 57.6 (corresponding to the above signal at *δ*_H_ 4.55 in the HSQC spectrum) and the significant downfield shift of the olefinic carbon signal from *δ*_C_ 114.4 to 153.0. In the HMBC spectrum of **4**, the protons of this oxygenated methylene demonstrated clear correlations to the downfield-shifted carbons C-20 at *δ*_c_ 153.0 and C-19 at *δ*_c_ 108.7, indicating its connection at C-20. No other significant differences were observed when comparing the NOESY spectra of **4** and **3**. Accordingly, the structure of **4** was established to be (5-(bis(3,8α-dimethyl-5-methylene-4,4α,5,6,7,8,8α,9-octahydronaphtho-[2,3-β]-furan-2-yl)methyl)furan-2-yl)methanol, trivially named 5-furanmethanol-2-methanetriyl-biatractylon.

Compound **5** gave the [M + H]^+^ ion at *m/z* = 739.4719 in its HR-APCI-MS spectrum, which was consistent with a molecular formula C_51_H_62_O_4_ possessing 21 degrees of unsaturation. As compared with **4**, compound **5** has six more degrees of unsaturation, 15 more carbons, and 18 more hydrogens in the molecular formula. Both its ^1^H and ^13^C-NMR spectra exhibit similar signal patterns to those of **4**, respectively, except for the occurrence of additional peaks which were attributed to atractylon moieties. Given that an atractylon moiety possesses 6 degrees of unsaturation, 15 carbons, and 19 hydrogens; it is implied that **5** should contain three atractylon moieties in its structure. Further analyses of the 1D and 2D NMR spectra confirmed the assignment, with the supporting data listed in Table 1. The methine proton (H-16) at *δ*_H_ 5.42 displayed clear HMBC correlations to the carbon pairs C-11/11′ at *δ*_C_ 115.1/115.1, C-12/12′ at *δ*_C_ 144.8/144.8, as well as C-17 at *δ*_C_ 151.5 and C-18 at *δ*_C_ 107.9, indicating two atractylon moieties were linked through this methine to the furan unit. The methylene protons (H-21) at *δ*_H_ 3.86 showed clear HMBC correlations to the carbon C-11″ at *δ*_C_ 114.9, C-12″ at *δ*_C_ 144.3, C-19 at *δ*_C_ 106.5, and C-20 at *δ*_C_ 151.4, revealing that the third atractylon moiety is linked through this methylene to the furan unit. On the basis of the above information, the structure of **5** was assigned as 2,2′-((5-((3,8α-dimethyl-5-methylene-4,4α,5,6,7,8,8α,9-octahydronaphtho-[2,3-β]-furan-2-yl)methyl)furan-2-yl)methylene)-bis(3,8α-dimethyl-5-methylene-4,4α,5,6,7,8,8α,9-octahydronaphtho-[2,3-β]-furan), trivially named furan-5-methanediyl-2-methanetriyl-triatractylon.

In addition to the aforementioned five new compounds, ten known compounds (**6**–**15**) were also isolated from the processed RAM in the present study (Figure 1). They were identified as 5-(hydroxymethyl)furfural (**6**) [16], 5-(Hydroxymethyl)-2-(dimethoxymethyl)furan (**7**) [17], 2,3-dihydro-3,5-dihydroxy-6-methyl-4-pyranone (**8**) [18], atractylon (**9**) [19], atractylenolide I (**10**) [20], atractylenolide II (**11**) [20], atractylenolide III (**12**) [20], atractylenolide VII (**13**) [21], γ-selinene (**14**) [22], and selina-4(14),7(11)-dien-8-one (**15**) [23] with comparing their spectroscopic data with those in the literature. Compound **7** is likely an artifact from **6** since methanol was used in the isolation process. Compound **8** was isolated from wheat [18], indicating that it might come from the wheat which was used during the process.

### 2.2. Proposed Mechanism for the Formation of Compounds ***1**–**5***

**Figure 2:**
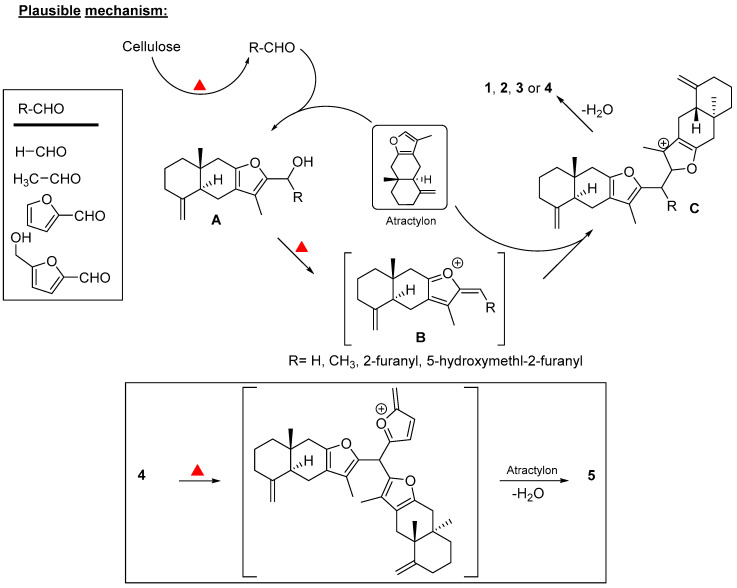
Proposed mechanism for the generation of compounds **1**–**5**.

To the best of our knowledge, this is the first report to delineate the possible role that processing plays in producing new chemical entities and impacting the chemical composition of traditional medicines. Five new compounds (**1**–**5**) were generated as a result of the processing of RAM by stir-frying with wheat bran. A plausible rationale for the formation of new atractylon derivatives (**1**–**5**) is outlined below. Both RAM and wheat bran contain fiber, polysaccharides, cellulose, resistant starch, inulin, lignins, and oligosaccharides [1,24]. Thermal processing of cellulose, hemicellulose, and other polysaccharides are known to produce significant amounts of diverse carbonyl compounds such as formaldehyde [25], acetaldehyde, furfural, 5-hydroxymethylfurfural, etc. [26]. Isolation of major quantities of both 5-(hydroxymethyl)furfural (**6**) and 5-(hydroxymethyl)-2-(dimethoxymethyl)furan (**7**) from the processed RAM in this study further supports the formation of carbonyl compounds. On the other hand, atractylon, the major sesquiterpene of *A. macrocephala* [15,27], can undergo electrophilic reactions with the pyrolytic aldehyde products. For example, as shown in the proposed plausible mechanism (Figure 2), atractylon would undergo electrophilic addition with RCHO (R can be hydrogen, methyl, 2-furanyl, or 5-hydroxymethylfuranyl) to yield a carbinol intermediate (**A**). Under pyrolytic conditions, the resulting carbinol would yield electrophilic species (**B**), which can be added to another molecule of atractylon to form an adduct (**C**). Further deprotonation would yield thermally stable adducts **1**–**4**. Moreover, compound **4** can undergo the same set of reactions (generation of electrophilic species, and addition to another molecule of atractylon and dehydration) to form compound **5**.

### 2.3. Biological Activities of Compounds ***1**–**5***

The new compounds (**1**–**5**) were evaluated for their cytotoxicity to a panel of selected mammalian cancer and noncancer cell lines (SK-MEL, KB, BT-549, SK-OV-3, LLC-PK1, and Vero cells). None of the compounds exhibited cytotoxic effects. All compounds were ineffective at inhibiting the growth of the pathogenic microorganisms including three fungi (*C. albicans*, *C. neoformans*, and *A. fumigates*) and five bacteria (*S. aureus*, methicillin-resistant *S. aureus*, *E. coli*, *P. aeruginosa*, and *M. intracellulare*). In addition, the in vitro antileishmanial activity testing revealed that the compounds were not effective against *L. donavani*. Even though the preliminary biological data of these interesting class of compounds are not encouraging, further biological studies on this class of compounds concerning other biological targets, as well as SAR and medicinal properties need to be further investigated. In addition to biological activities, probing the comparative physicochemical properties of these polymeric compounds against monomeric atractylon might provide additional scientific rationale behind the traditional processes associated with polyherbal formulations.

## 3. Materials and Methods

### 3.1. General Experimental Procedures

UV and IR spectra were obtained on a HP 8452A UV-Vis spectrometer (Hewlett-Packard, Palo Alto, CA, USA) and an Cary 630 FTIR spectrometer (Agilent Technologies, Santa Clara, CA, USA), respectively. High-resolution mass spectra were obtained on an Agilent TOF LC/MS spectrometer equipped with an atmospheric pressure chemical ionization (APCI) source and operated with the Analyst QS 1.1 software for data acquisition and processing. NMR spectra were acquired on an Agilent DD2-500 NMR spectrometer with a OneNMR probe at 500 MHz for ^1^H and 125 MHz for ^13^C using the pulse programs provided by the Agilent Vnmrj 4.0 software. Silica gel (J. T. Baker, 40 μm for flash chromatography) and Sephadex LH-20 were purchased from Fisher Scientific Co. (Waltham, MA, USA), and used for column chromatographic separations. Biotage Isolera^TM^ Prime flash chromatography system (Biotage Co, Charlotte, NC, USA) was used for further separation and purification. TLC was performed on silica gel 60 GF 254 plates (Millipore Sigma, Burlington, MA, USA) with the TLC spots being observed at 254 nm, followed by spraying with 1% vanillin-H_2_SO_4_ derivatization reagent.

### 3.2. Plant Material and Preparation

The rhizomes of *A. macrocephala* were purchased from the Harbin Medicine Group Shiyitang Chinese Herbal Medicine Pharmaceutical Co. (Harbin City, Heilongjiang, China), and authenticated by Prof. Zhenyue Wang at the Heilongjiang University of Chinese Medicine. A voucher specimen (No. 20150013) was deposited in the Natural Products Laboratory, the School of Pharmacy of Heilongjiang University of Chinese Medicine. The wheat bran, produced in Heilongjiang, was bought from the Harbin Medicinal Materials market.

Processing of the rhizomes of *A. macrocephala* (2.5 kg) was conducted in the Processing Lab of the Experimental Training Center in the Heilongjiang University of Chinese Medicine. Following the processing protocol in the Chinese Pharmacopeia, the rhizomes were cut into slices and air-dried to reach a constant weight. The wheat bran was first stir-fried in a cauldron with a temperature around 170 °C until smoke appeared. The rhizome slices were then added into the cauldron with a ratio of the rhizome to wheat bran of 1:4 (*w/w*), the mixture was stir-fried approximately 26 mins until the slices turned to a yellow-brown color. Next, the processed rhizome slices were separated from the wheat bran by a sifter.

### 3.3. Extraction and Isolation

The processed slices of rhizomes (2 kg) of *A. macrocephala* were powdered and extracted with methanol (10 L × 3 times) using ultrasonic extraction for 1 hour for each cycle. After the solvent was evaporated under reduced pressure, a total of 206.8 g of crude extract was obtained. The obtained extract was suspended in water (3 L) and successively partitioned with hexane (3 L × 3 times) and EtOAc (3 L × 3 times) to yield 44.4 g and 44.0 g partition fractions, respectively, after removal of solvents in vacuo. The hexane fraction (44.0 g) was subjected to a silica gel column (CC) (820 g, 10 × 80 cm) for chromatographic fractionation, eluting with a hexane-CHCl_3_ solvent system by stepwise increasing the polarity. The fractions were collected and combined based on TLC analysis to afford 18 fractions (Fr.1–18). Fr.5 (77.4 mg) was chromatographed on a Sephadex LH-20 column (45 g, 2 × 80 cm), eluted with CHCl_3_-MeOH (2.5:1) to yield compound **1** (12.1 mg). Fr.4 (77.1 mg) was first separated on a Biotage chromatographic system (SNAP KP-Sil 100 g, eluting with hexane-CH_2_Cl_2_ solvent system in increasing CH_2_Cl_2_ concentration from 15% to 80%), then purified by Sephadex LH-20 (45 g, 2 × 80 cm, eluting with CHCl_3_-MeOH 2:1) and silica gel column chromatography (CC) (9.25 g, 1.5× 40 cm, stepwise eluting with hexane-CH_2_Cl_2_) to give **2** (6.2 mg). Fr.8 (239.8 mg) was chromatographed on a silica gel column (120 g, 4 × 80 cm) eluted with MeOH-CHCl_3_ (1:1), the targeted fraction was further purified using a Sephadex LH-20 column (45 g, 2 × 80 cm), eluting with CHCl_3_-MeOH while gradually increasing the polarity, then further purified on a silica gel column (8.9 g, 2 × 40 cm) eluting with hexane-CH_2_Cl_2_ to yield **3** (13.5 mg). Fr.15 (4.20 g) was first chromatographed on a silica gel column (120g, 4× 80 cm) eluting with hexane-EtOAc in increasing polarity, and then further purified on a Sephadex LH-20 column (45 g, 2 × 80 cm) eluting with CHCl_3_-MeOH (2:1) to yield **4** (7.4 mg). Fr.10 (297.1 mg) was treated similarly to that of Fr. 4 to obtain **5** (9.6 mg).

### 3.4. Characterization of Compounds ***1**–**5***

*Bis(3,8*α*-dimethyl-5-methylene-4,4*α*,5,6,7,8,8*α*,9-octahydronaphtho-[2,3-*β*]-furan-2-yl)methane* (**1**), (methylene-biatractylon), white amorphous powder. UV (MeOH): λ_max_ (logε) = 231 (3.95) nm. IR (ART): *V*_max_ = 2926.0, 2849.5, 1641.9, 1440.6, 1378.1, 1231.9, 1250.5, 1136.8, 889.0, 758.5 cm^−1^. ^1^H-NMR and ^13^C-NMR data see Table 1. HR-APCI-MS: *m/z* = 445.3134 [M + H]^+^ (calc’d. for C_31_H_41_O_2_ = 445.3106).

*2,2′-(Ethane-1,1-diyl)-bis(3,8*α*-dimethyl-5-methylene-4,4*α*,5,6,7,8,8*α*,9-octahydronaphtho-[2,3-*β*]-furan)* (**2**), (ethylidene-biatractylon), white amorphous powder. UV (MeOH): λ_max_ (logε) = 228 (4.02) nm. IR (ART): *V*_max_ = 2926.0, 2847.7, 1641.9, 1440.6, 1377.3, 1138.7, 889.0, 758.5 cm^−1^. ^1^H-NMR and ^13^C-NMR data see Table 1. HR-APCI-MS: *m/z* = 459.3267 [M + H]^+^ (calc’d. for C_32_H_43_O_2_ = 459.3262).

*2,2′-(furan-2-ylmethylene)-bis(3,8*α*-dimethyl-5-methylene-4,4*α*,5,6,7,8,8*α*,9-octahydronaphtho-[2,3-*β*]-furan)* (**3**) (furan-2-methanetriyl--biatractylon), white amorphous powder. UV (MeOH): λ_max_ (logε) = 229 (4.12) nm. IR (ART): *V*_max_ = 2924.1, 2849.5, 1641.9, 1377.3, 1440.6, 1340.0, 1250.5, 1231.9, 1181.6, 1136.8, 899.0, 762.2, 730.6 cm^−1^. ^1^H-NMR and ^13^C-NMR data see Table 1. HR-APCI-MS: *m/z* = 511.3221 [M + H]^+^ (calc’d. for C_35_H_43_O_3_ = 511.3212).

*(5-(bis(3,8*α*-dimethyl-5-methylene-4,4*α*,5,6,7,8,8*α*,9-octahydronaphtho-[2,3-*β*]-furan-2-yl)methyl)furan-2-yl)methanol* (**4**) (5-furanmethanol-2-methanetriyl-biatractylon), white amorphous powder. UV (MeOH): λ_max_ (logε) = 230 (4.17) nm. IR (ART): *V*_max_ = 2924.1, 2845.8, 1641.9, 1440.6, 1377.3, 1250.5, 1233.7, 1136.8, 1013.8, 889.0, 786.5, 758.5 cm^−1^. ^1^H-NMR and ^13^C-NMR data see Table 1. HR-APCI-MS: *m/z* = 541.3299 [M + H]^+^ (calc’d. for C_36_H_45_O4 = 541.3318).

*2,2′-((5-((3,8*α*-dimethyl-5-methylene-4,4*α*,5,6,7,8,8*α*,9-octahydronaphtho-[2,3-*β*]-furan-2-yl)methyl)furan-2-yl)methylene)bis(3,8*α*-dimethyl-5-methylene-4,4*α*,5,6,7,8,8*α*,9-octahydronaphtho-[2,3-*β*]-furan)* (**5**) (furan-5-methanediyl-2-methanetriyl-triatractylon), white amorphous powder. UV (MeOH): λ_max_ (logε) 230 (4.30) nm. IR (ART): *V*_max_ = 2953.9, 2924.1, 2853.3, 1459.3, 1377.3, 1272.9, 1136.8, 1123.8, 1073.5, 1015.7, 889.0 cm^−1^. ^1^H-NMR and ^13^C-NMR data see Table 1. HR-APCI-MS: *m/z* = 739.4719 [M + H]^+^ (calc’d. for C_51_H_63_O_4_ = 739.4726).

The NMR, MS, UV, and IR spectra of above five compounds are presented in the Supplementary Materials, which are available free of charge via the internet at https://www.mdpi.com/journal/molecules.

### 3.5. Biological Activity Assays

#### 3.5.1. Cytotoxicity Assay

Cytotoxic activity was determined against four human cancer cell lines (SK-MEL, KB, BT-549, and SKOV-3) and two noncancerous kidney cell lines (LLC-PK1 and Vero) as described earlier [28]. All cell lines were obtained from the American Type Culture Collection (ATCC, Rockville, MD, USA). Each assay was performed in 96-well tissue culture-treated microplates. Cells were seeded at a density of 25,000 cells/well and incubated for 24 h. Samples at different concentrations were added, and cells were again incubated for 48 h. At the end of incubation, the cell viability was measured using a tetrazolium dye (WST-8) which was converted to a water-soluble formazan product. The absorbance was measured at 450 nm and the percent viability of sample treated cells was calculated in comparison to the vehicle-treated cells. Doxorubicin was used as a positive control, while DMSO was used as the negative (vehicle) control.

#### 3.5.2. Antimicrobial Assays

Isolates were tested against a panel of 8 pathogenic organisms including three fungi (*Candida albicans* ATCC 90028, *Cryptococcus neoformans* ATCC 90113, and *Aspergillus fumigates* ATCC 204305) and five bacteria (*Staphylococcus aureus* ATCC 29213, methicillin-resistant *S. aureus* ATCC 33591, *Escherichia coli* ATCC 35218, *Pseudomonas aeruginosa* ATCC 27853, and *Mycobacterium intracellulare* ATCC 23068). Microorganisms were obtained from the American Type Culture Collection. The assays were performed at the National Center for Natural Products Research (NCNPR), at the University of Mississippi as a part of the antimicrobial screening program following a previously reported method [29]. Drug controls, ciprofloxacin for bacteria and amphotericin B for fungi, were included in each assay.

#### 3.5.3. Antileishmanial Assay

Compounds were tested in vitro for their ability to inhibit *Leishmania donovani*, employing the assay described by Jain et al. [30]. Amphotericin B was included as the drug control for *L. donovani*.

## 4. Conclusions

Pao-Zhi (frying and cooking of herbs) is an ancient pharmaceutic technique in TCM to facilitate the use of herbal medicines for specific clinic needs [31]. Traditionally, most Chinese herbal medicines undergo elaborate processing in order to become ingredients that are prescribed or utilized in the manufacturing of TCM proprietary drugs [32]. Although the practice of processing has a long history, the underlying mechanisms largely remain unclear for most Chinese herbal medicines. In the present study, through the characterization of chemical profiles coupled with NMR metabolomics approach, the chemical changes resulting from the traditional processing protocol associated with RAM preparation were investigated. For the first time, five new chemical adducts, which were formed during processing *A. macrocephala* with wheat bran, were isolated and their structures were identified. The findings allowed us to gain valuable insights into the chemical reactions which occur during the processing procedures. Processed RAM is widely used in various formulations of TCM drugs and health care products. Stir-frying with wheat bran is one of the most widely used traditional processing methods for RAM in TCM [12]. Although the processed RAM is listed as an item in the Chinese Pharmacopoeia, there are currently no modern standardized processing protocols or quality control standards for the processed RAM products. The findings of this study may provide useful information for developing such standards with a scientific basis. Furthermore, as the change of chemical profile will inevitably influence the associated pharmacological properties of processed RAM, further investigations of the bio-activities of the newly generated compounds are needed.

## Figures and Tables

**Table 1:** ^1^H-(500 MHz) and ^13^C-(125 MHz) NMR data of compounds **1**–**5** (recorded in CDCl_3_, ppm).

No. *	1		2		3		4		5	
	δ_C_	δ_H_	δ_C_	δ_H_	δ_C_	δ_H_	δ_C_	δ_H_	δ_C_	δ_H_
1, 1′, (1′′)	42.0 (t)	1.47, 1.68 (m, 4H)	41.9/42.0 (t)	1.49, 1.67 (m, 4H)	42.0 (t)	1.47, 1.66 (m, 4H)	41.9 (t)	1.48, 1.65 (m, 4H)	42.0 (t)	1.47, 1.67 (m, 6H)
2, 2′, (2′′)	23.6 (t)	1.57, 1.63 (m, 4H)	23.6 (t)	1.56, 1.63 (m, 4H)	23.6 (t)	1.55, 1.64 (m, 4H)	23.6 (t)	1.53, 1.63 (m, 4H)	23.6 (t)	1.54, 1.64 (m, 6H)
3, 3′, (3′′)	37.3 (t)	2.02, 2.37 (m, 4H)	37.4 (t)	2.02, 2.37 (m, 4H)	37.3 (t)	2.02, 2.38 (m, 4H)	37.3 (t)	2.02, 2.38 (m, 4H)	37.3/36.7 (t)	2.02, 2.37 (m, 6H)
4, 4′, (4′′)	150.0 (s)		150.0 (s)		149.9 (s)		149.9 (s)		149.9/150.0 (s)	
5, 5′, (5′′)	45.7 (d)	2.10 (m, 2H)	45.7/45.8 (d)	2.10 (m, 2H)	45.7 (d)	2.10 (m, 2H)	45.6/45.7 (d)	2.09 (m, 2H)	45.6/45.7 (d)	2.10 (m, 3H)
6, 6′, (6′′)	21.2 (t)	2.25, 2.32 (m, 4H)	21.1 (t)	2.25, 2.32 (m, 4H)	21.1 (t)	2.27, 2.34 (m, 4H)	21.1 (t)	2.25, 2.32 (m, 4H)	21.1 (t)	2.25, 2.32 (m, 6H)
7, 7′, (7′′)	116.6 (s)		116.6 (s)		116.8 (s)		116.8 (s)		116.8/116.7 (s)	
8, 8′, (8′′)	146.9 (s)		146.6 (s)		147.6 (s)		147.6 (s)		147.3/147.0 (s)	
9, 9′, (9′′)	39.2 (t)	2.41 (m, 4H)	39.2/39.3 (t)	2.42 (m, 4H)	39.2 (t)	2.42 (m, 4H)	39.2 (t)	2.40 (m, 4H)	39.2/39.1 (t)	2.40/2.39 (m, 6H)
10, 10′, (10′′)	36.7 (s)		36.7 (s)		36.7 (s)		36.7 (s)		36.7/36.4 (s)	
11, 11′, (11′′)	114.3 (s)		113.0 (s)		115.2 (s)		115.2 (s)		115.1/114.9 (s)	
12, 12′, (12′′)	150.0 (s)		149.1 (s)		144.7 (s)		144.5 (s)		144.8/144.3 (s)	
13, 13′, (13′′)	8.0 (q)	1.87 (s, 6H)	7.8/8.0 (q)	1.76/1.82 (s, 6H)	7.8/7.9 (q)	1.80/1.81 (s, 6H)	7.8/7.9 (q)	1.79/1.80 (s, 6H)	7.8/7.9/8.0 (q)	1.74/1.76/1.82 (s, 9H)
14, 14′, (14′′)	17.6 (q)	0.76 (s, 6H)	17.6/17.7 (q)	0.75/0.77 (s, 6H)	17.6/17.7 (q)	0.75/0.76 (s, 6H)	17.6/17.7 (q)	0.75/0.76 (s, 6H)	17.6/17.7 (q)	0.75/0.76 (s, 9H)
15, 15′, (15′′)	107.1 (t)	4.68, 4.84 (d, 4H)	107.1 (t)	4.68, 4.84 (d, 4H)	107.1 (t)	4.68, 4.84 (d, 4H)	107.2 (t)	4.67, 4.83 (d, 4H)	107.1/107.2 (t)	4.67, 4.83 (d, 6H)
16	23.9 (t)	3.82 (s, 2H)	30.1 (d)	4.17 (q, 1H)	36.1 (d)	5.47 (s, 1H)	36.2 (d)	5.44 (s, 1H)	36.2 (d)	5.42 (s, 1H)
17			18.1 (q)	1.57 (d, 3H)	153.1 (s)		153.2 (s)		151.5 (s)	
18					107.2 (d)	6.07 (d, 1H)	108.1 (d)	6.02 (d, 1H)	107.9 (d)	5.93 (d, 1H)
19					110.2 (d)	6.30 (dd, 1H)	108.7 (d)	6.20 (d, 1H)	106.5 (d)	5.89 (d, 1H)
20					141.4 (d)	7.35 (d, 1H)	153.0 (s)		151.4 (s)	
21							57.6 (t)	4.55 (s, 2H)	25.8 (t)	3.86 (s, 2H)

## Supplementary Materials

The supplementary information is available online. Figure S1.1: ^1^H-NMR spectrum of compound **1**, Figure S1.2: ^13^C-NMR spectrum of compound **1**, Figure S1.3: HSQC spectrum of compound **1**, Figure S1.4: COSY spectrum of compound **1**, Figure S1.5: HMBC spectrum of compound **1**, Figure S1.6: HR-APCI-MS spectrum of compound **1**, Figure S1.7: IR spectrum of compound **1**, Figure S1.8: UV spectrum of compound **1**, Figure S2.1: ^1^H-NMR spectrum of compound **2**, Figure S2.2: ^13^C-NMR spectrum of compound **2**, Figure S2.3: HSQC spectrum of compound **2**, Figure S2.4: COSY spectrum of compound **2**, Figure S2.5: HMBC spectrum of compound **2**, Figure S2.6: HR-APCI-MS spectrum of compound **2**, Figure S2.7: IR spectrum of compound **2**, Figure S2.8: UV spectrum of compound **2**, Figure S3.1: ^1^H-NMR spectrum of compound **3**, Figure S3.2: ^13^C-NMR spectrum of compound **3**, Figure S3.3: HSQC spectrum of compound **3**, Figure S3.4: COSY spectrum of compound **3**, Figure S3.5: HMBC spectrum of compound **3**, Figure S3.6: HR-APCI-MS spectrum of compound **3**, Figure S3.7: IR spectrum of compound **3**, Figure S3.8: UV spectrum of compound **3**, Figure S4.1: ^1^H-NMR spectrum of compound **4**, Figure S4.2: ^13^C-NMR spectrum of compound **4**, Figure S4.3: HSQC spectrum of compound **4**, Figure S4.4: COSY spectrum of compound **4**, Figure S4.5: HMBC spectrum of compound **4**, Figure S4.6: HR-APCI-MS spectrum of compound **4**, Figure S4.7: IR spectrum of compound **4**, Figure S4.8: UV spectrum of compound **4**, Figure S5.1: ^1^H-NMR spectrum of compound **5**, Figure S5.2: ^13^C-NMR spectrum of compound **5**, Figure S5.3: HSQC spectrum of compound **5**, Figure S5.4: COSY spectrum of compound **5**, Figure S5.5: HMBC spectrum of compound **5**, Figure S5.6: HR-APCI-MS spectrum of compound **5**, Figure S5.7: IR spectrum of compound **5**, Figure S5.8: UV spectrum of compound **5**.
