# Supplementary material for: Newly Generated Atractylon Derivatives in Processed Rhizomes of Atractylodes macrocephala Koidz
Source: Molecules. 2020 Dec 13;25(24):5904. doi: 10.3390/molecules25245904 (PMC7763829; doi:10.3390/molecules25245904)

SUPPORTING INFORMATION

Newly Generated Atractylon Derivatives in Processed Rhizomes of *Atractylodes macrocephala* Koidz

Chunmei Zhai[a,b], Jianping Zhao[b], Amar G. Chittiboyina[b], Yonghai Meng[a,b], Mei Wang[b, c], Shabana I. Khan[b], Ikhlas A. Khan[a,b,d,*]

[a] Heilongjiang University of Chinese Medicine, Harbin 150040, Heilongjiang. P. R. China

[b] National Center for Natural Products Research, School of Pharmacy, University of Mississippi, MS 38677, USA

[c] Natural Products Utilization Research Unit, Agricultural Research Service, U.S. Department of Agriculture, University, Mississippi 38677, USA

[d] Division of Pharmacognosy, Department of BioMolecular Sciences, School of Pharmacy, University of Mississippi, MS 38677, USA

*Corresponding author
Prof. Ikhlas A. Khan
Director, National Center for Natural Products Research, Thad Cochran Research Center, P.O. Box 1848, University of Mississippi, University, MS 38677, USA
Email: ikhan@olemiss.edu
Telephone: 01-662 915 7821, Fax: 01-662 915 7062

Table of Contents:

Figure S1.1. ^{1}H NMR spectrum of compound **1** ……………………………………….…….....S4

Figure S1.2. ^{13}C-NMR spectrum of compound **1** ……………...……………………………….. S4

Figure S1.3. HSQC spectrum of compound **1** ..………………………………………….......S5

Figure S1.4. COSY spectrum of compound **1** ……………………………………………….S5

Figure S1.5. HMBC spectrum of compound **1** ……………………………………………....S6

Figure S1.6. HR-APCI-MS spectrum of compound **1**……………………..…………...... S6

Figure S1.7. IR spectrum of compound **1** ……….…………………………..…………......S7

Figure S1.8. UV spectrum of compound **1** ……….…………………………..………….....S7

Figure S2.1. ^{1}H NMR spectrum of compound **2** ……………………………………….…….....S8

Figure S2.2. ^{13}C-NMR spectrum of compound **2** ……………...…………………………….…...S8

Figure S2.3. HSQC spectrum of compound **2** ..………………………………………….......S9

Figure S2.4. COSY spectrum of compound **2** ……………………………………………….S9

Figure S2.5. HMBC spectrum of compound **2** ………………………………………………S10

Figure S2.6. HR-APCI-MS spectrum of compound **2**……………………..…………......S10

Figure S2.7. IR spectrum of compound **2** ……….…………………………..…………......S11

Figure S2.8. UV spectrum of compound **2** ……….…………………………..………….....S11

Figure S3.1. ^{1}H NMR spectrum of compound **3** ……………………………………….……....S12

Figure S3.2. ^{13}C-NMR spectrum of compound **3** ……………...……………… ……………..S12

Figure S3.3. HSQC spectrum of compound **3** ..……………………………………………....S13

Figure S3.4. COSY spectrum of compound **3** ………………………………………………S13

Figure S3.5. HMBC spectrum of compound **3** ……………………………………………...S14

Figure S3.6. HR-APCI-MS spectrum of compound **3**……………………..…………......S14

Figure S3.7. IR spectrum of compound **3** ………..…………………………..………….......S15

Figure S3.8. UV spectrum of compound **3** ………..…………………………..…………......S15

Figure S4.1. ^{1}H NMR spectrum of compound **4** …………………… ………………..……......S16

Figure S4.2. ^{13}C-NMR spectrum of compound **4** ……………...……………………… ……..S16

Figure S4.3. HSQC spectrum of compound **4** ..……………………………………………….S17

Figure S4.4. COSY spectrum of compound **4** ………………………………………………S17

Figure S4.5. HMBC spectrum of compound **4** ………………………………………………S18

Figure S4.6. HR-APCI-MS spectrum of compound **4**………………………..…………......S18

Figure S4.7. IR spectrum of compound **4** ………..…………………………..………….......S19

Figure S4.8. UV spectrum of compound **4** ………..…………………………..…………......S19

Figure S5.1. ^{1}H NMR spectrum of compound **5** ………………………………….… S20

Figure S5.2. ^{13}C-NMR spectrum of compound **5** ……………...…………… ………………..S20

Figure S5.3. HSQC spectrum of compound **5** ..…………………………………………......S21

Figure S5.4. COSY spectrum of compound **5** ……………………………………………….S21

Figure S5.5. HMBC spectrum of compound **5** ……………………………………………...S22

Figure S5.6. HR-APCI-MS spectrum of compound **5**………………………..…………......S22

Figure S5.7. IR spectrum of compound **5** ………..…………………………..………….......S23

Figure S5.8. UV spectrum of compound **5** ………..…………………………..…………......S23

Figure S1.1. ^{1}H NMR spectral of compound **1**

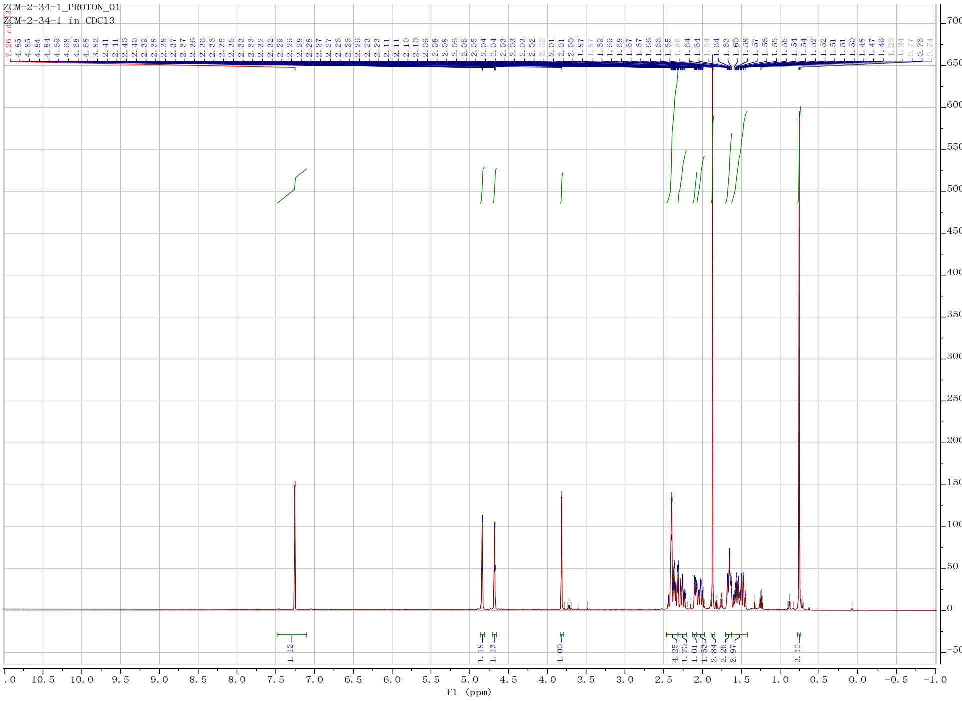

Figure S1.2. ^{13}C-NMR spectral of compound **1**

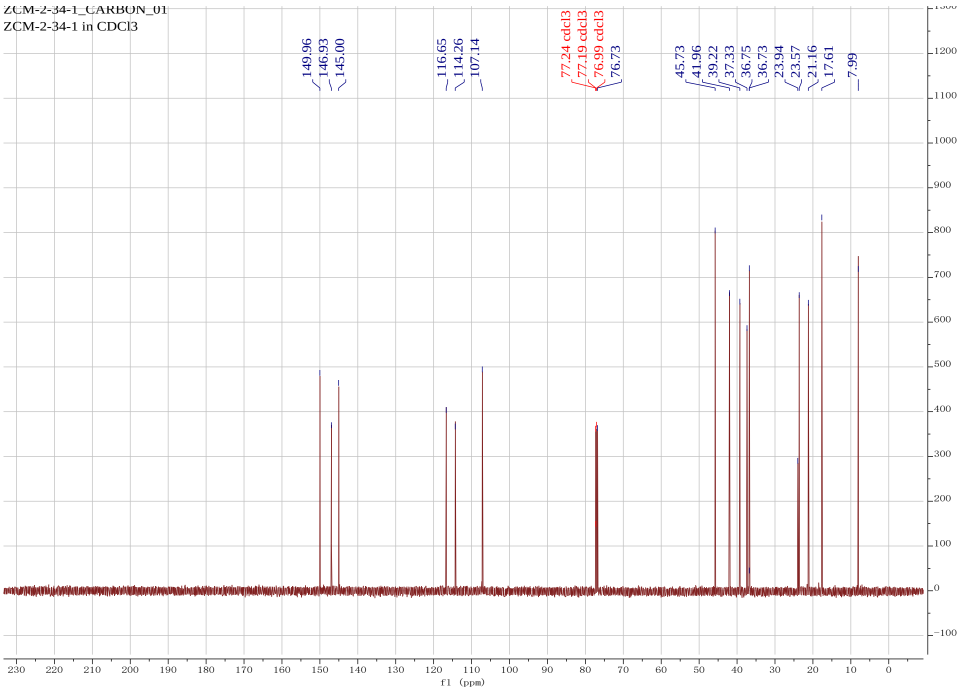

Figure S1.3. HSQC spectrum of compound **1**

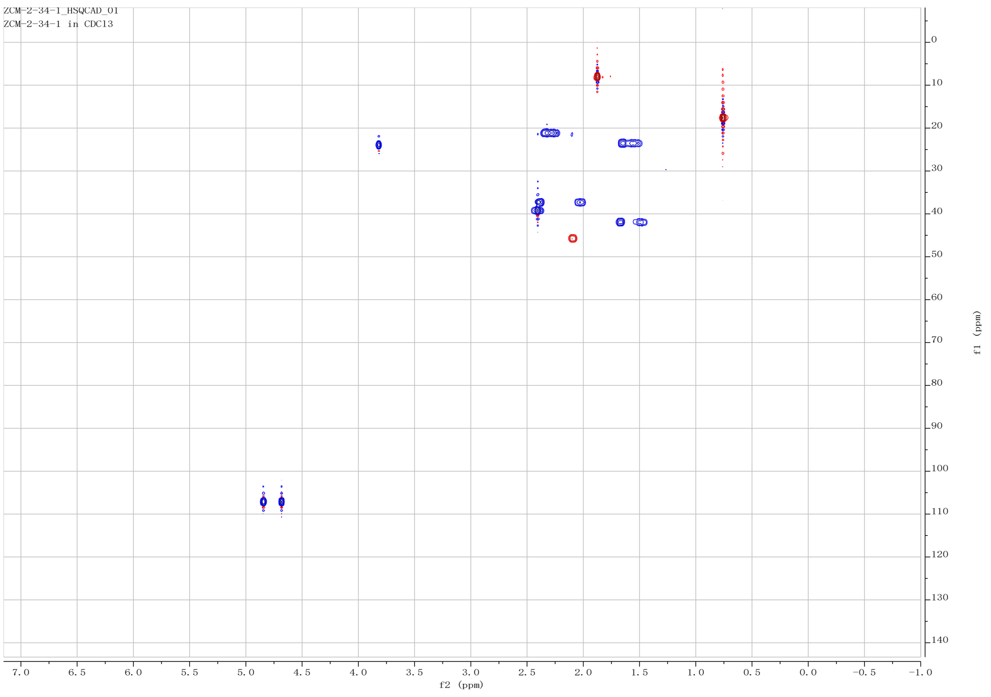

Figure S1.4. COSY spectrum of compound **1**

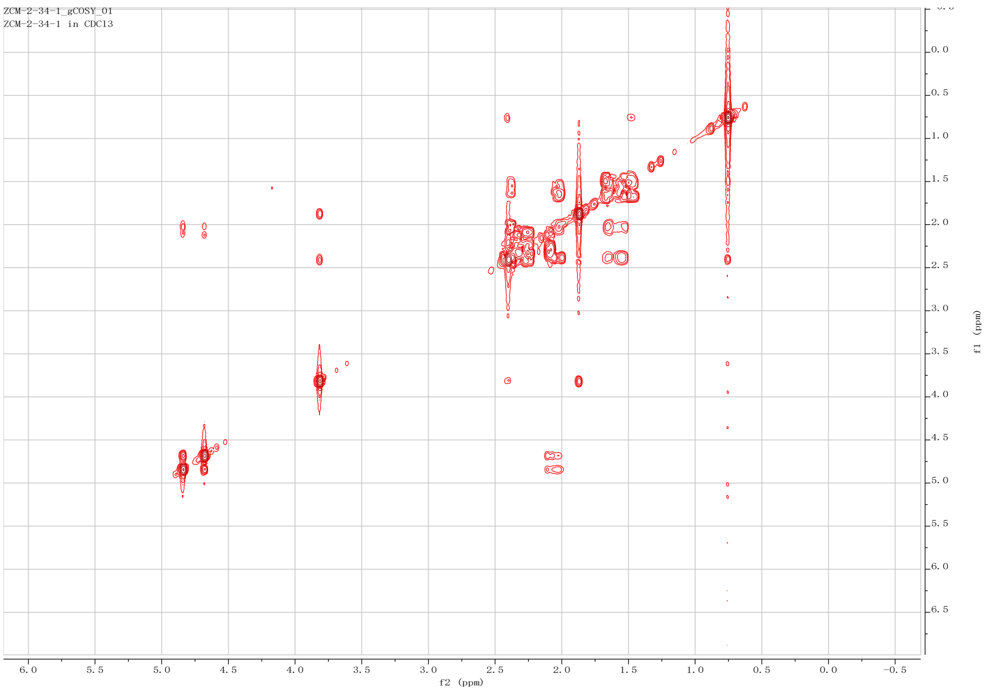

Figure S1.5. HMBC spectrum of compound **1**

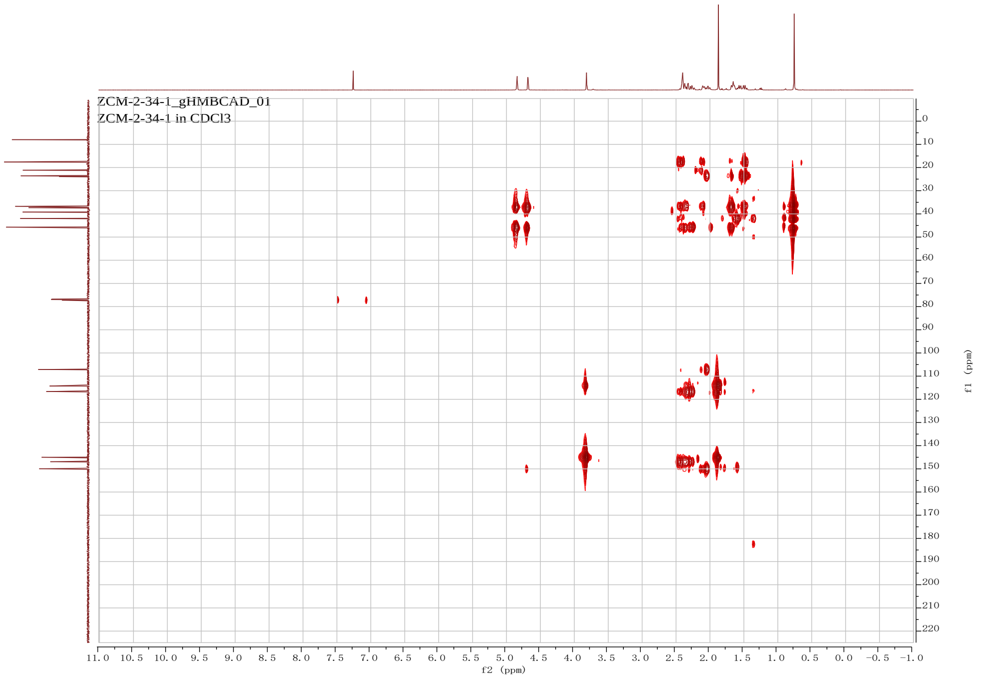

Figure S1.6. HR-APCI-MS spectrum of compound **1**

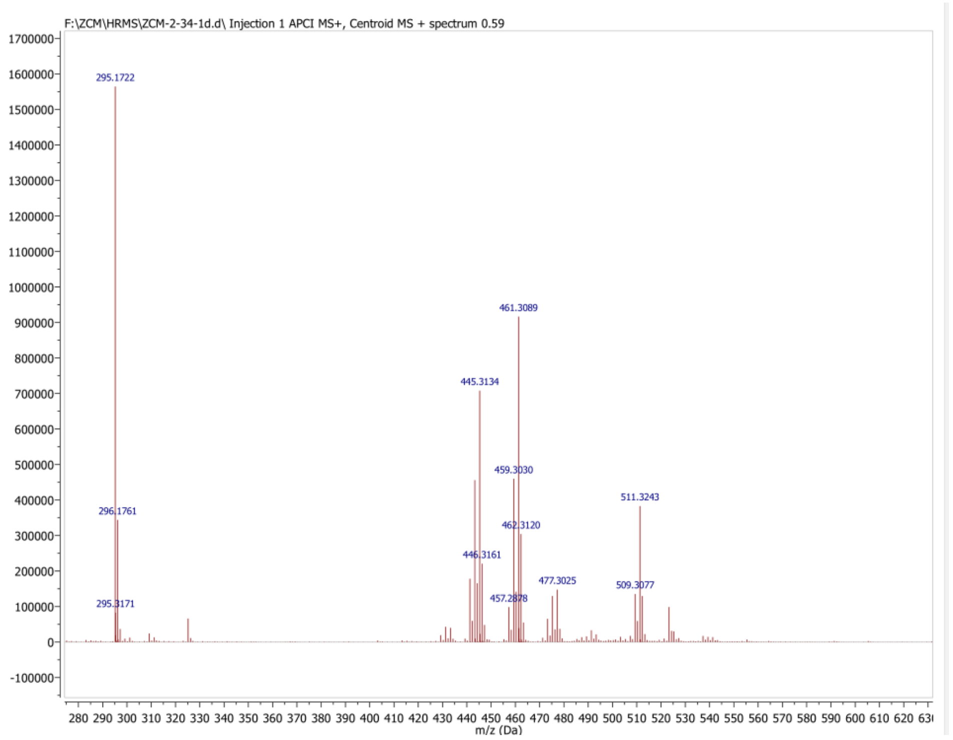

Figure S1.7. IR spectrum of compound **1**

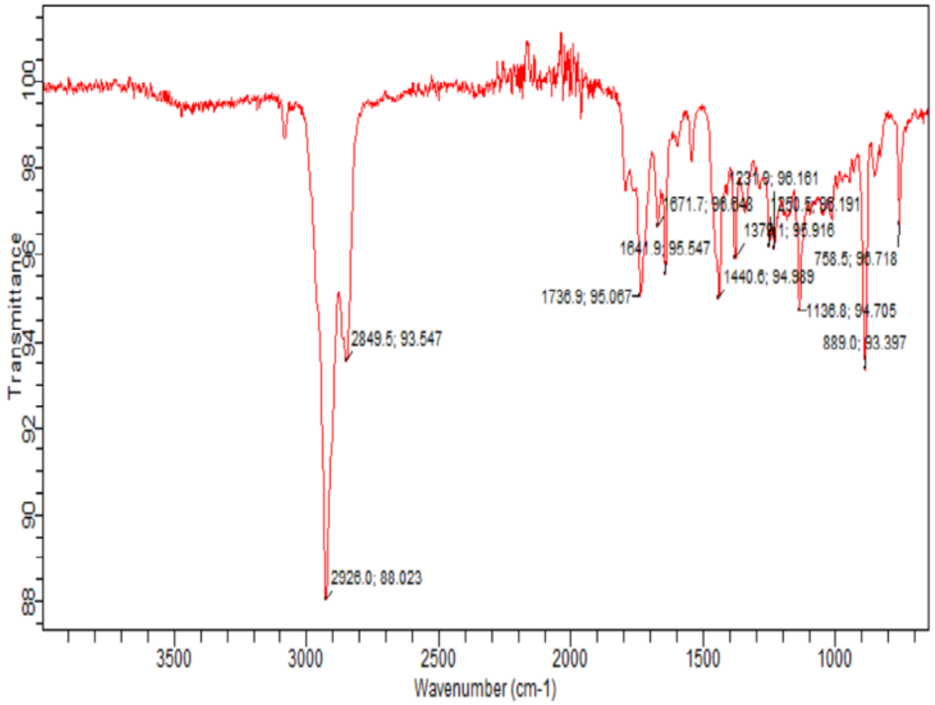

Figure S1.8. UV spectrum of compound **1**

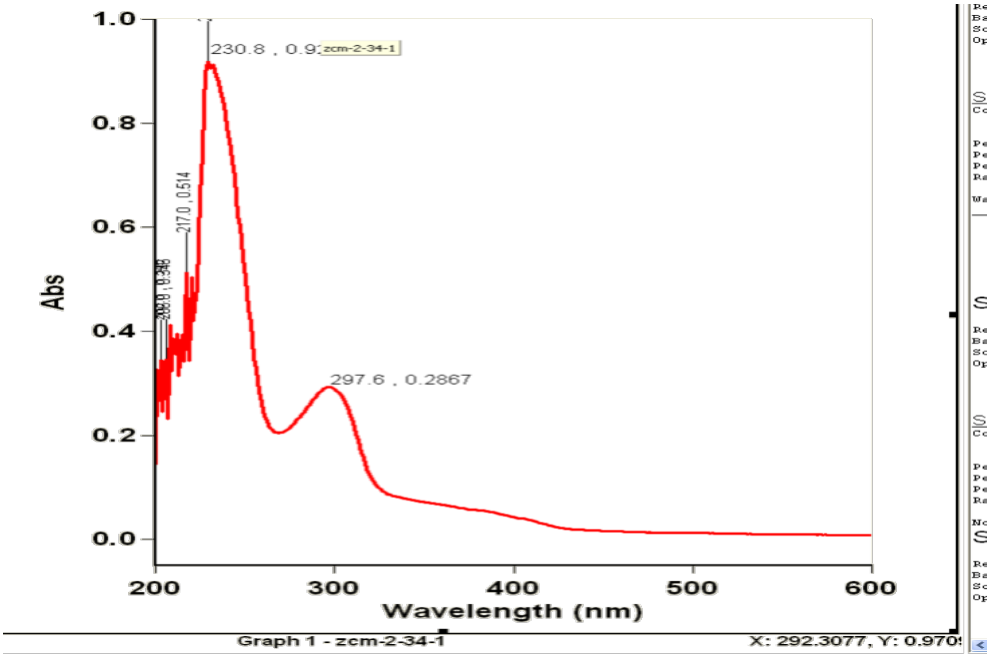

Figure S2.1. ^{1}H NMR spectral of compound **2**

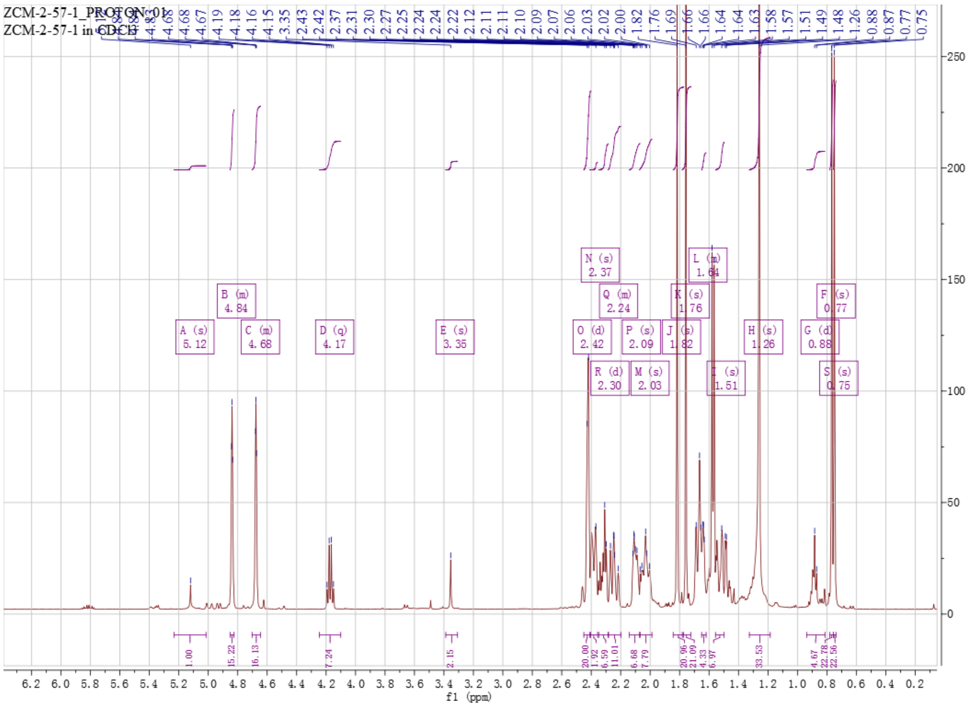

Figure S2.2. ^{13}C-NMR spectral of compound **2**

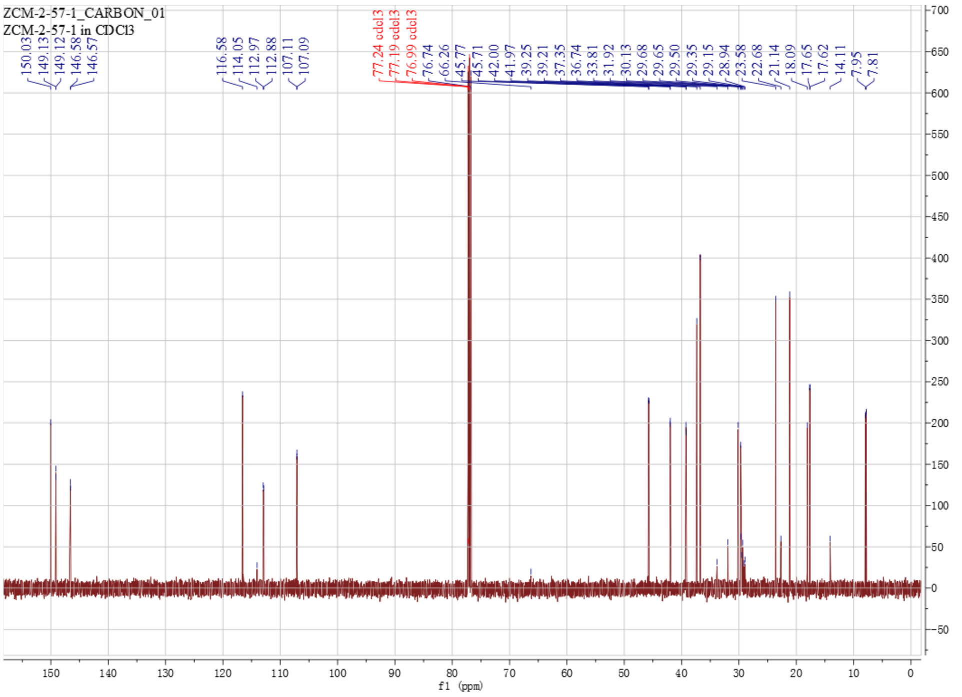

Figure S2.3. HSQC spectrum of compound **2**

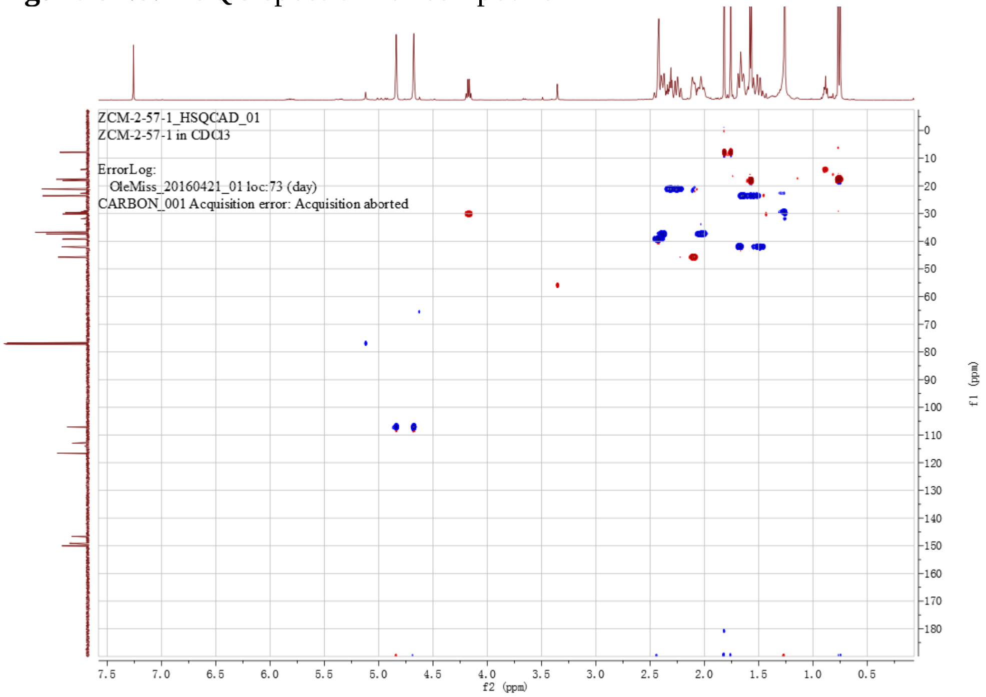

Figure S2.4. COSY spectrum of compound **2**

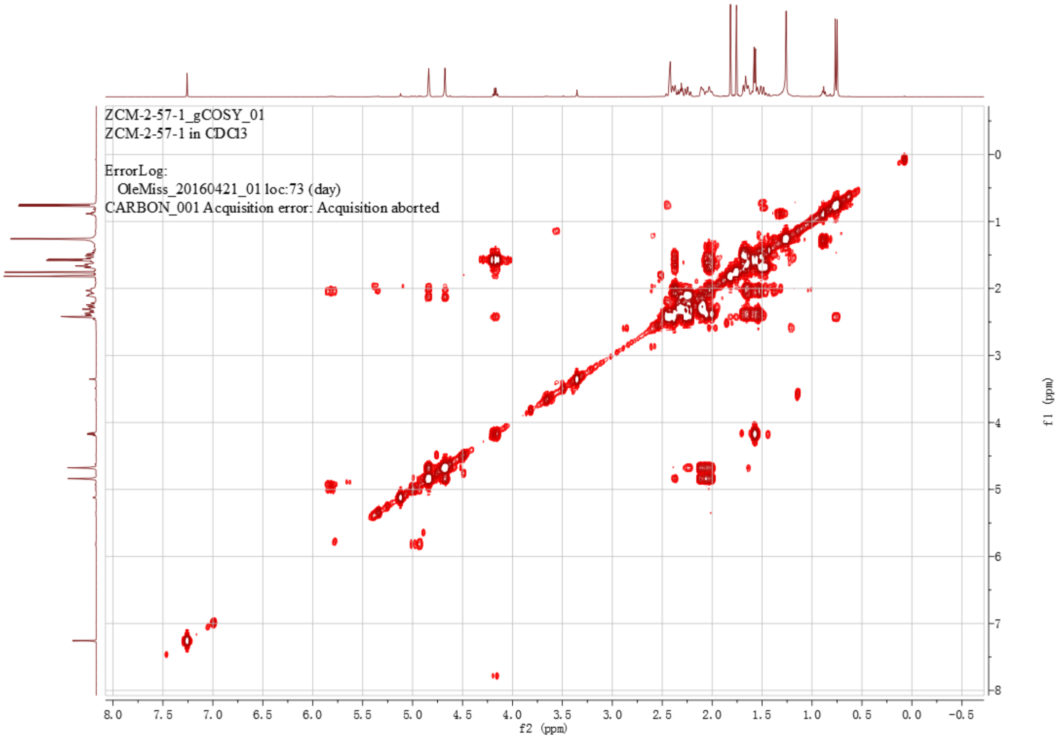

Figure S2.5. HMBC spectrum of compound **2**

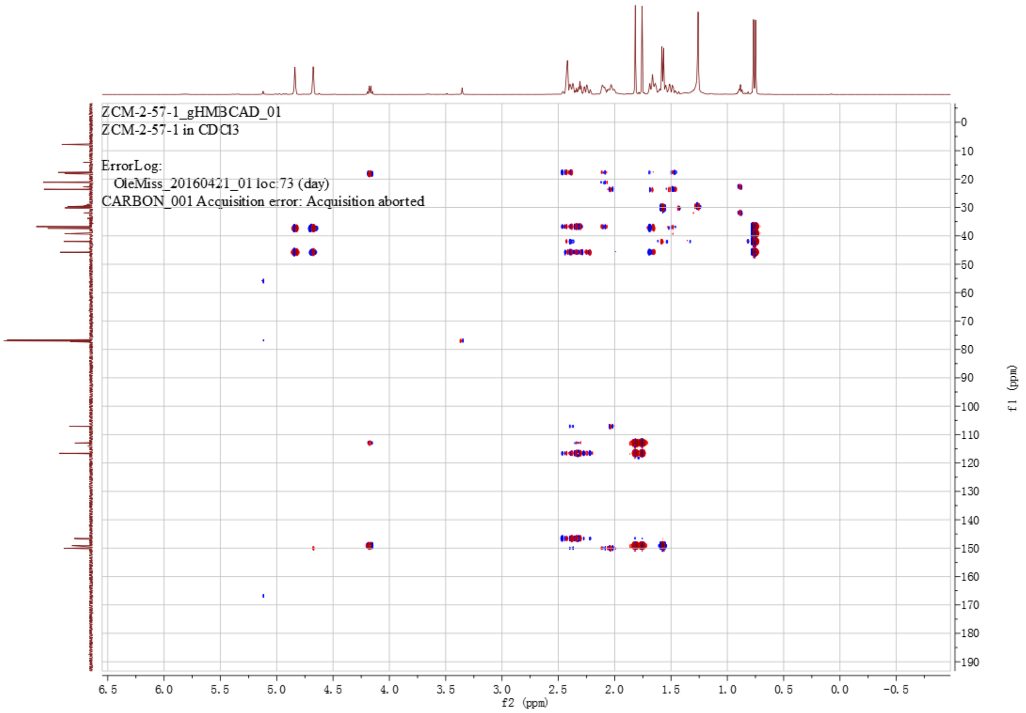

Figure S2.6. HR-APCI-MS spectrum of compound **2**

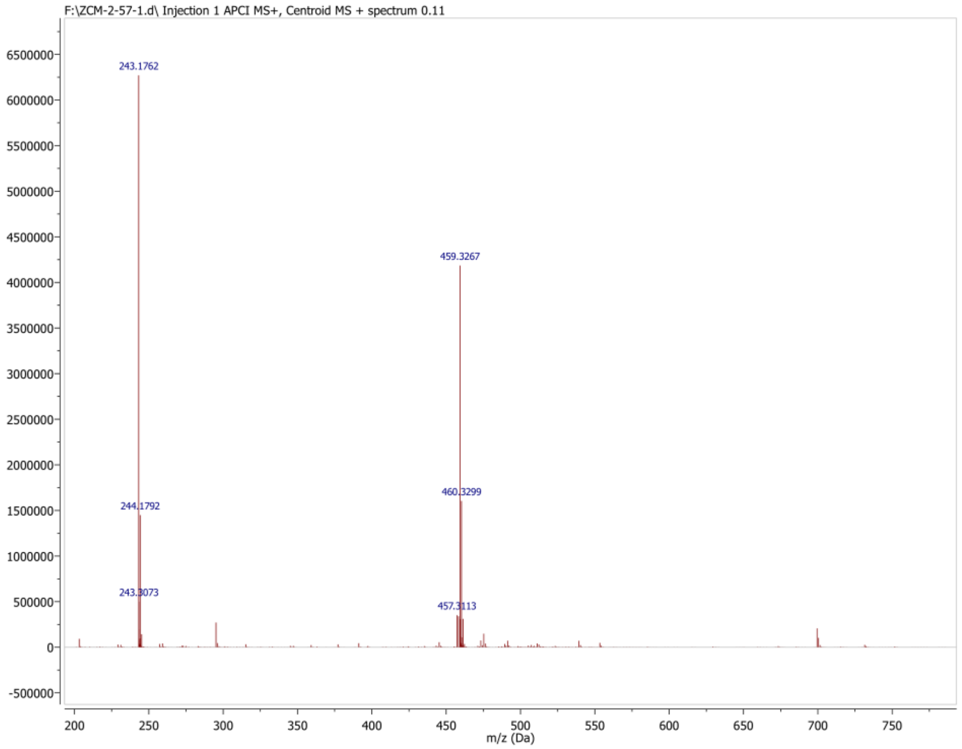

Figure S2.7. IR spectrum of compound **2**

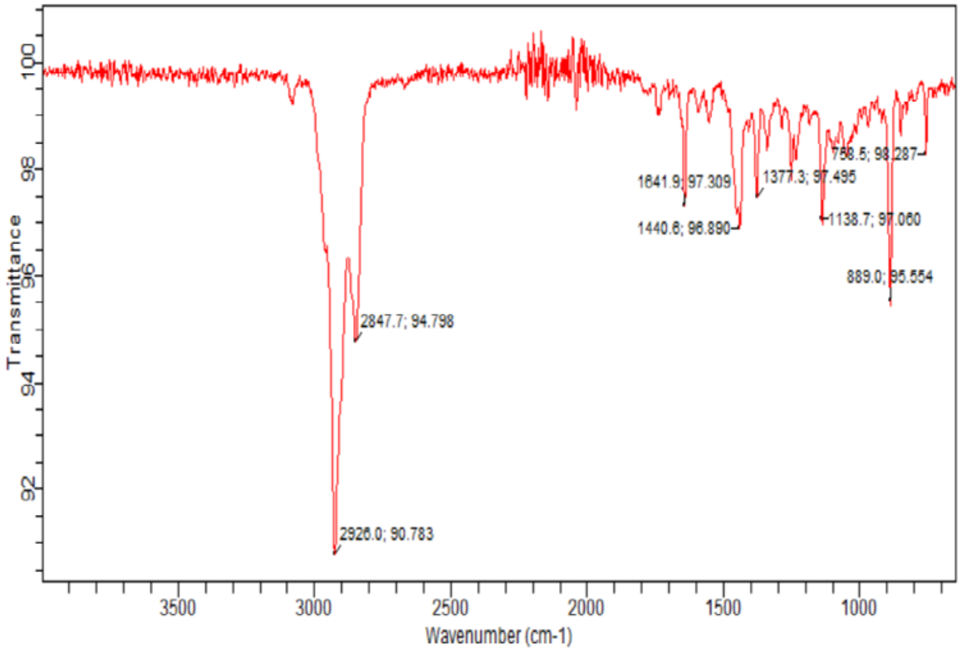

Figure S2.8. UV spectrum of compound **2**

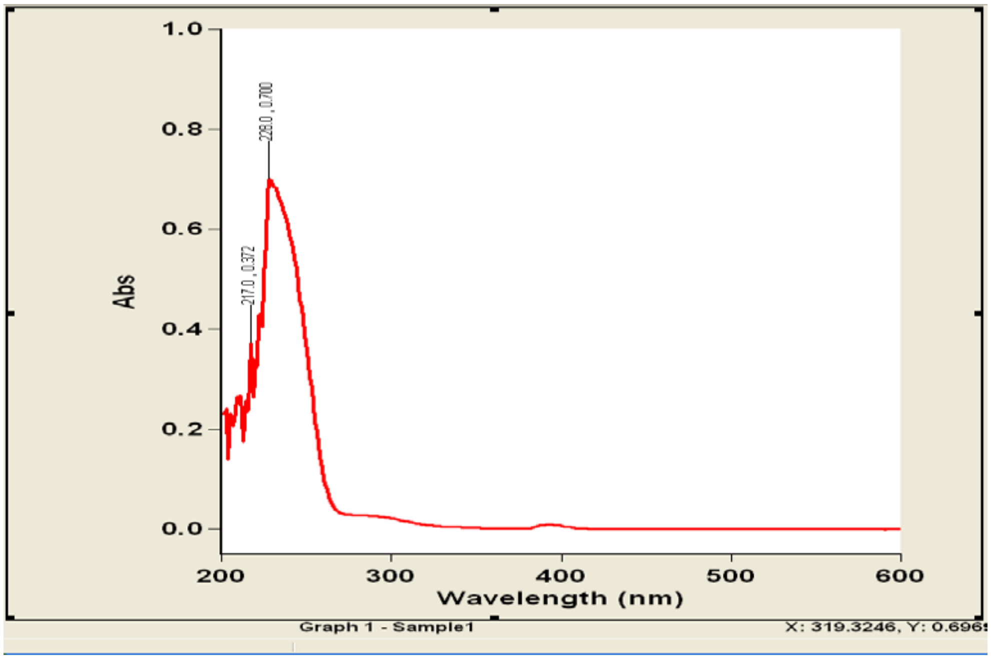

Figure S3.1. ^{1}H NMR spectral of compound **3**

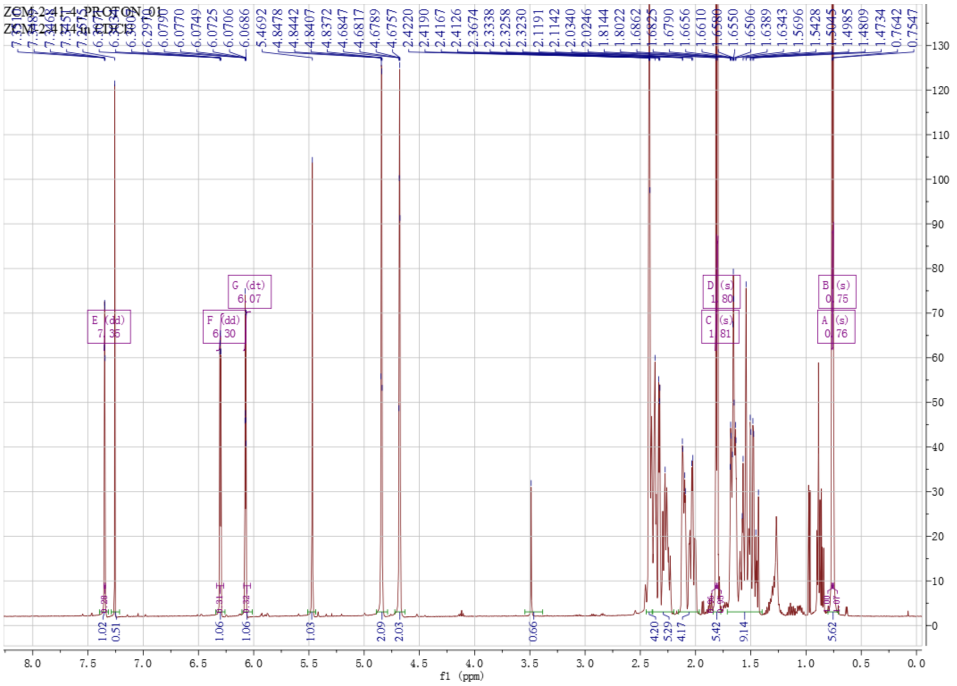

Figure S3.2. ^{13}C-NMR spectral of compound **3**

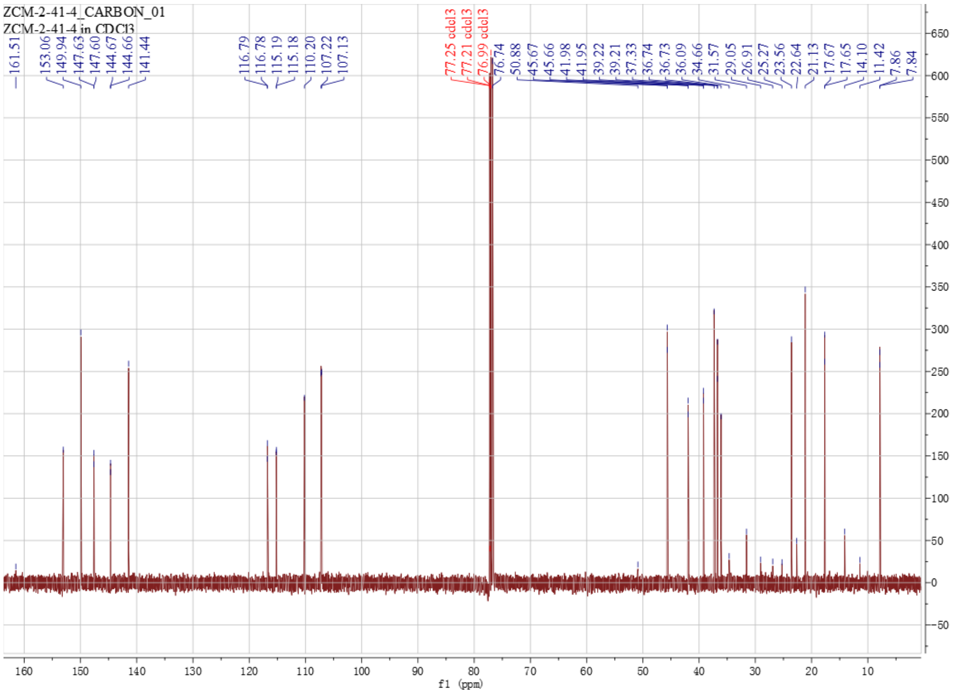

Figure S3.3. HSQC spectrum of compound **3**

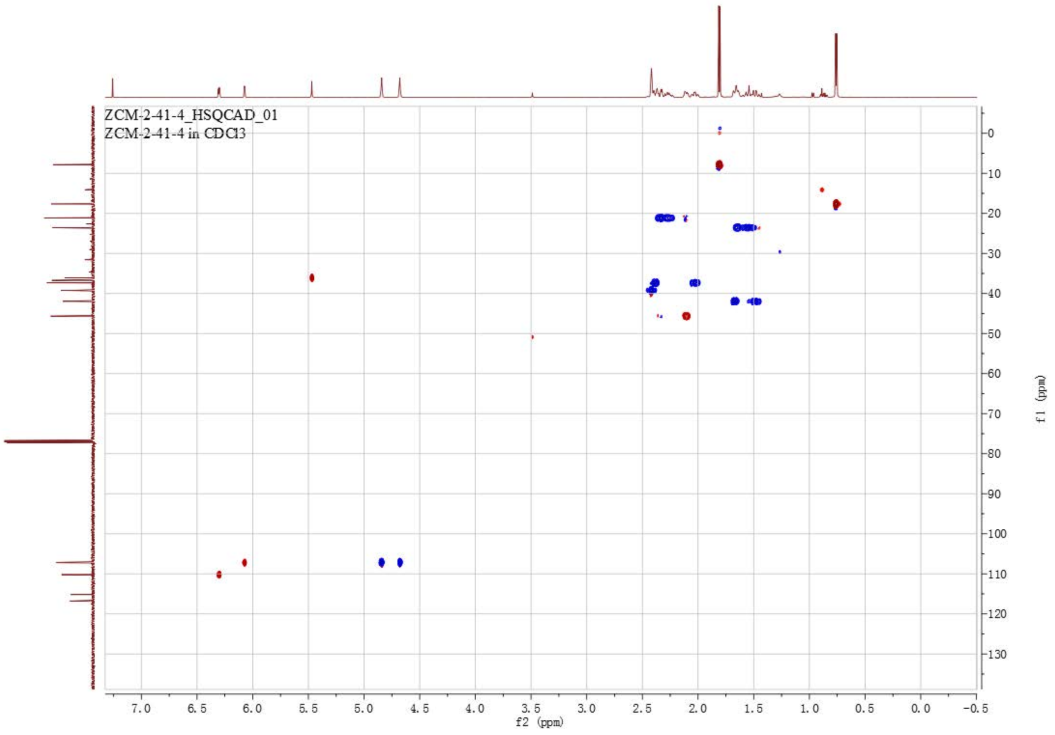

Figure S3.4. COSY spectrum of compound **3**

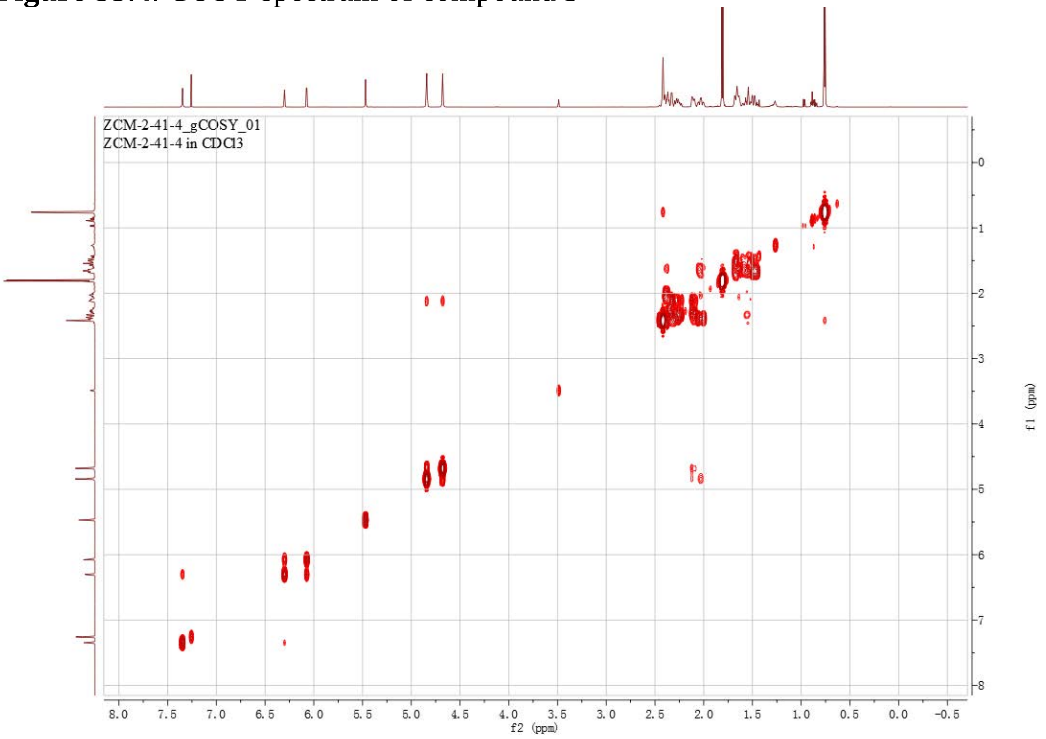

Figure S3.5. HMBC spectrum of compound **3**

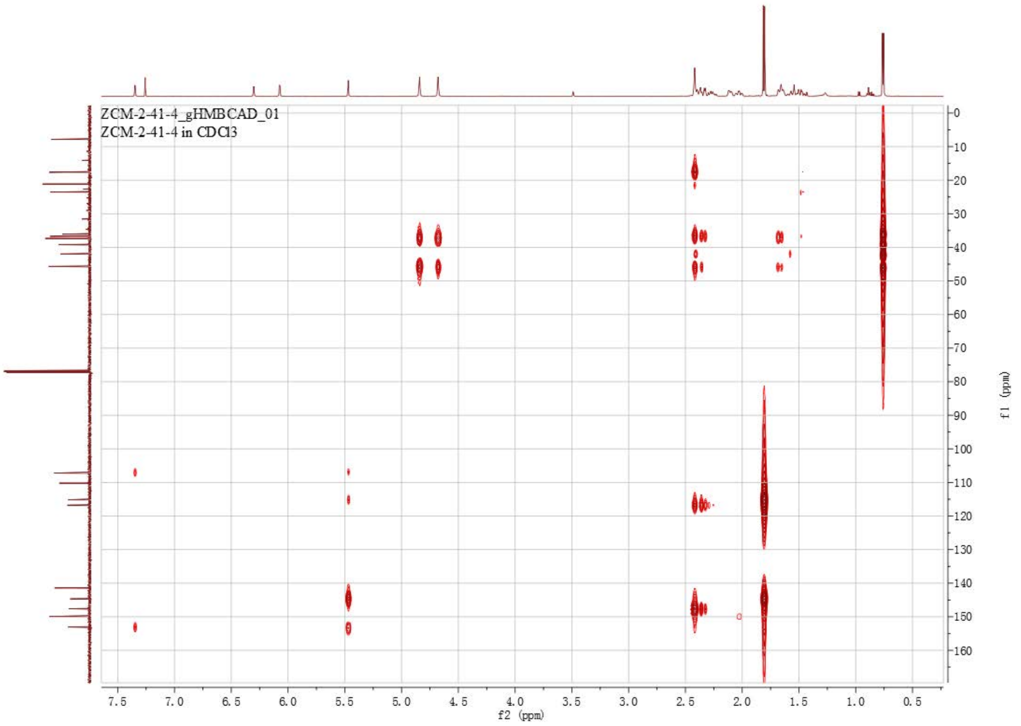

Figure S3.6. HR-APCI-MS spectrum of compound **3**

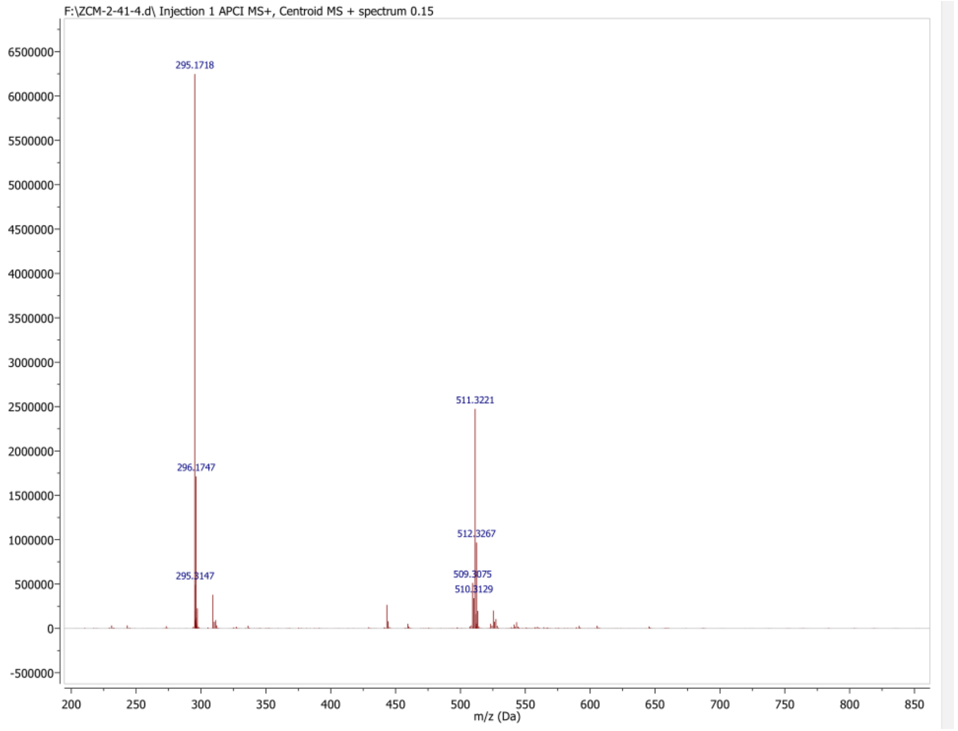

Figure S3.7. IR spectrum of compound **3**

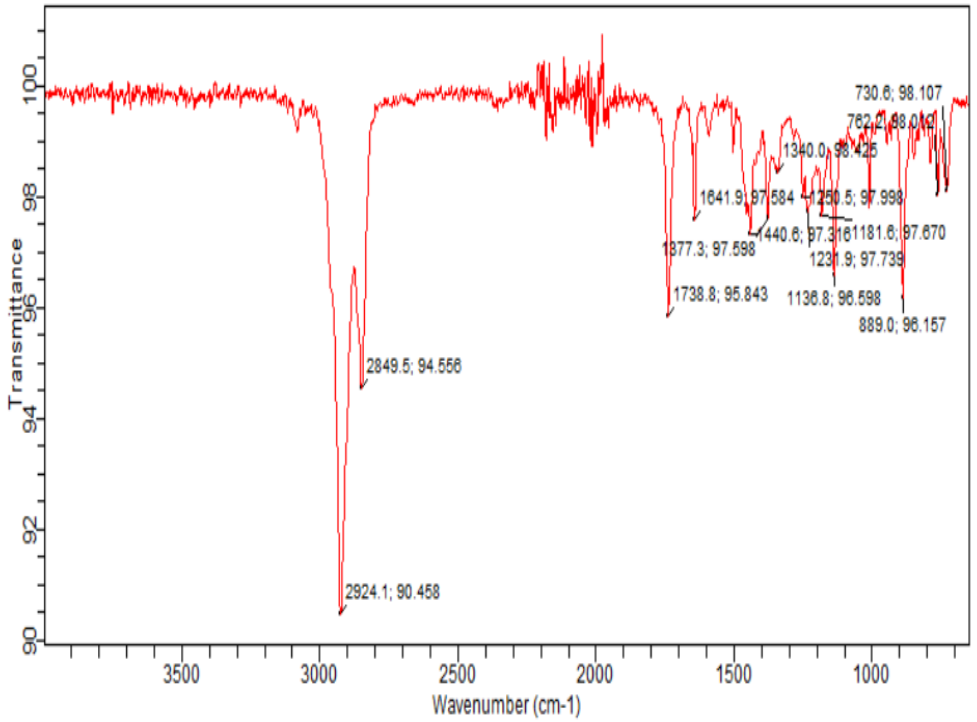

Figure S3.8. UV spectrum of compound **3**

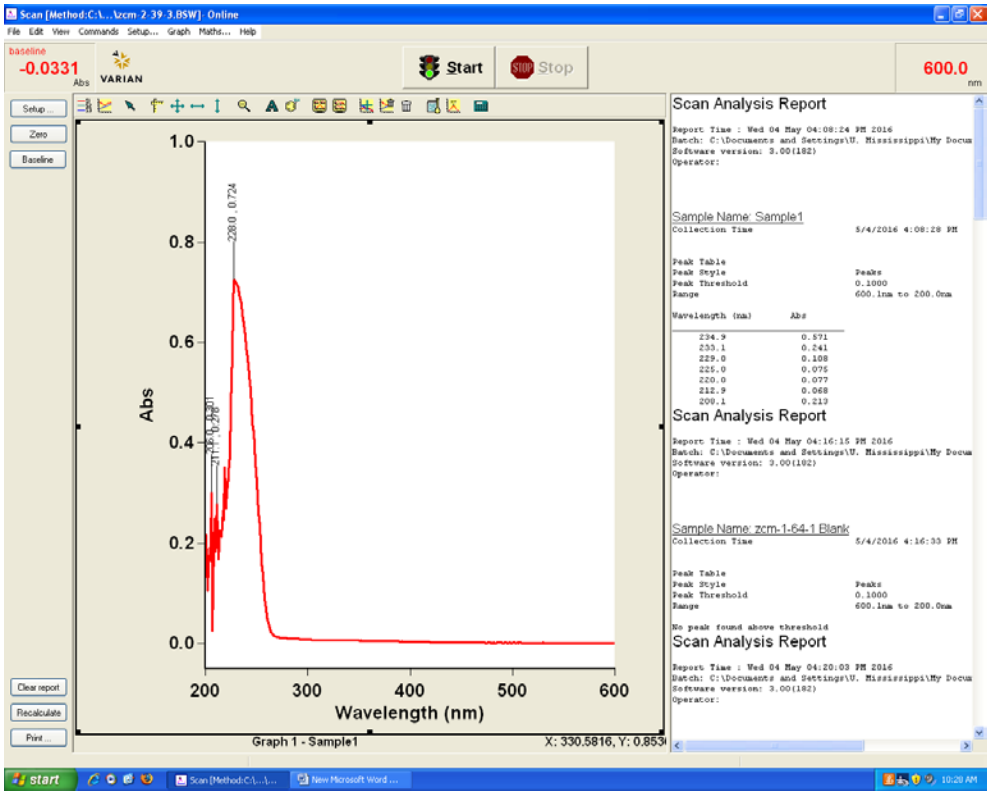

Figure S4.1. ^{1}H NMR spectral of compound **4**

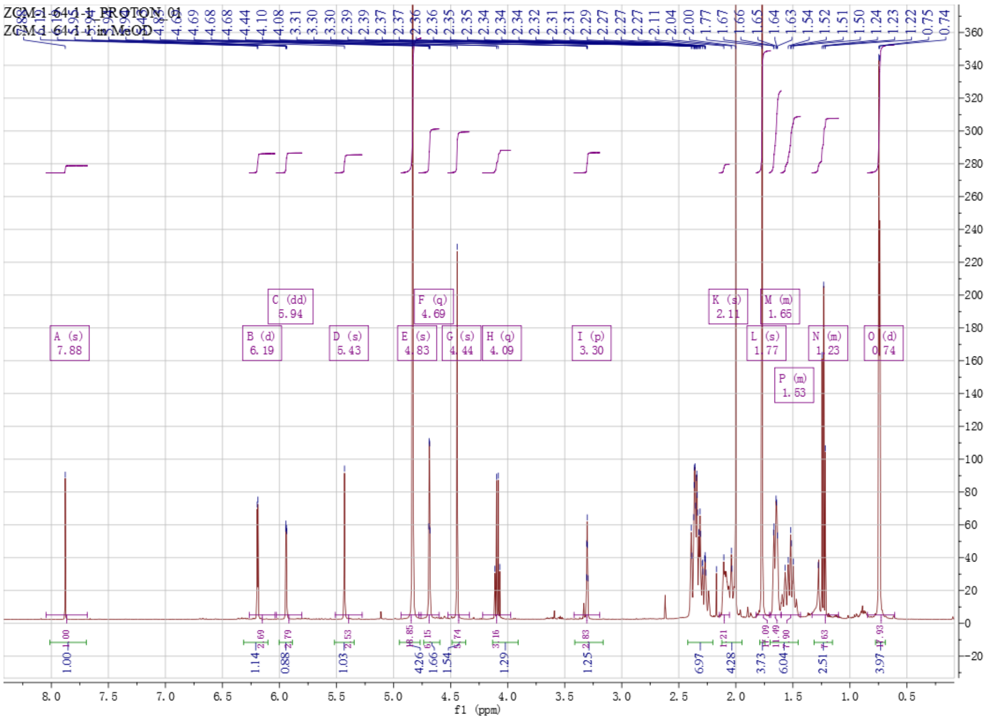

Figure S4.2. ^{13}C-NMR spectral of compound **4**

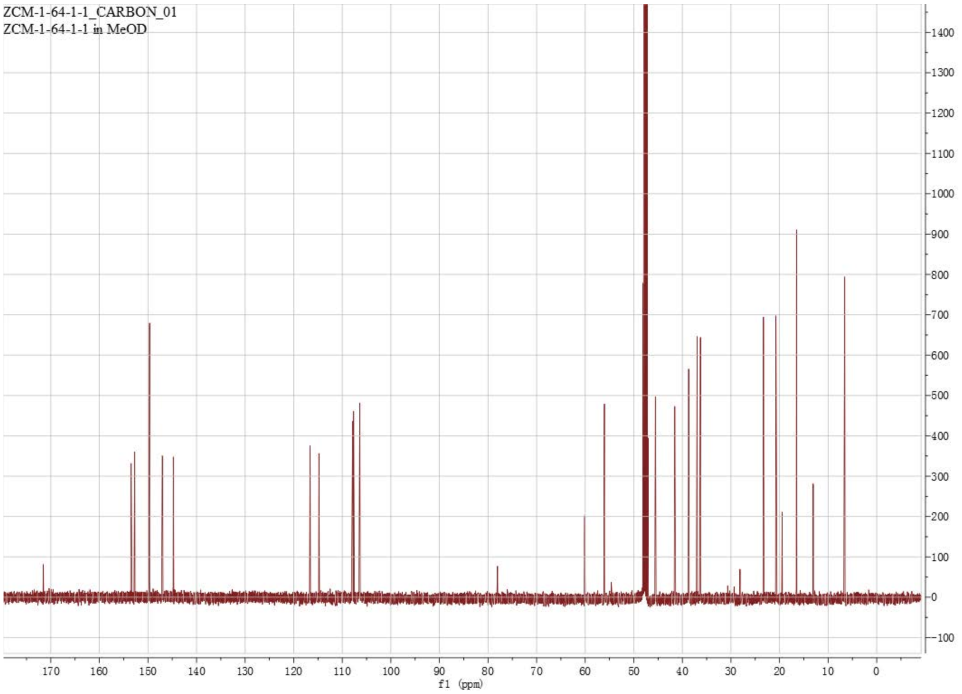

Figure S4.3. HSQC spectrum of compound **4**

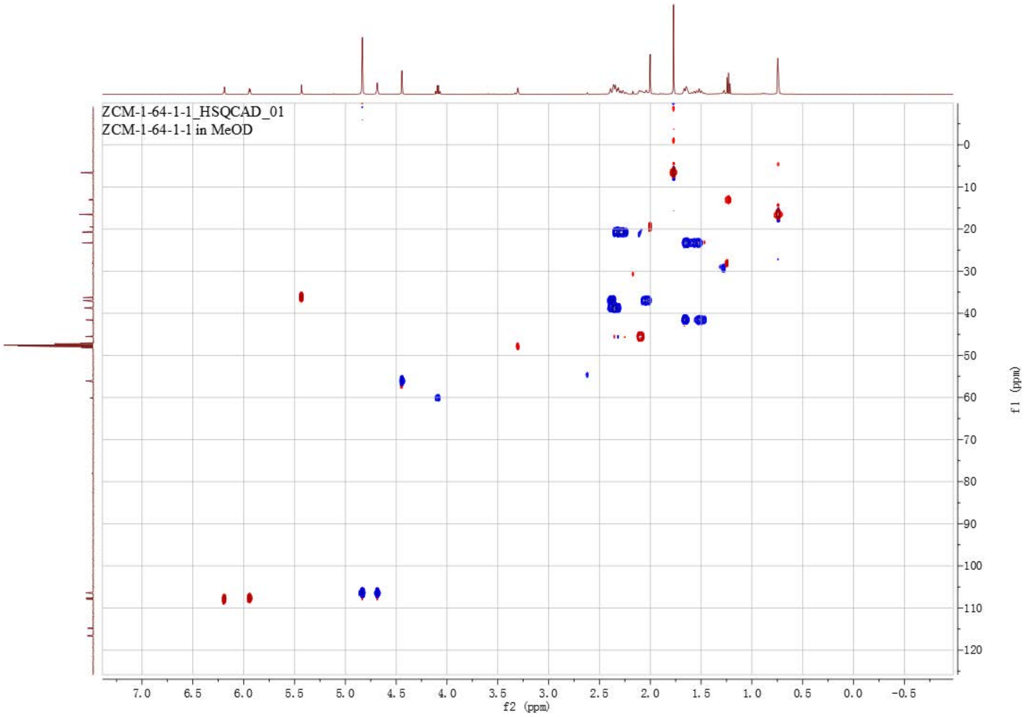

Figure S4.4. COSY spectrum of compound **4**

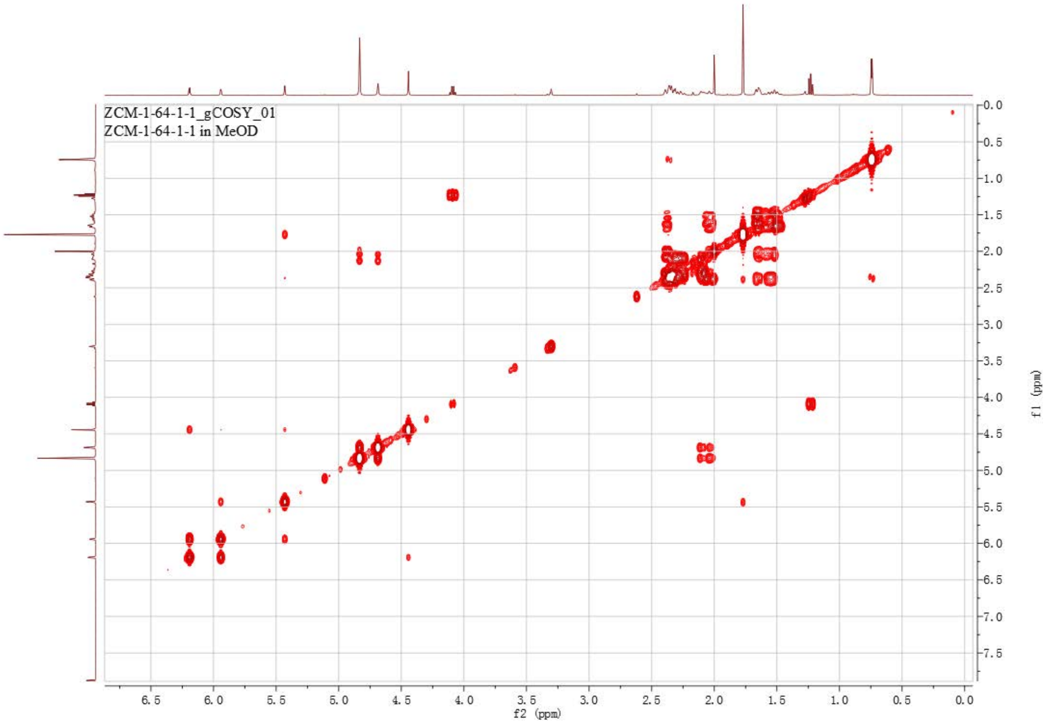

Figure S4.5. HMBC spectrum of compound **4**

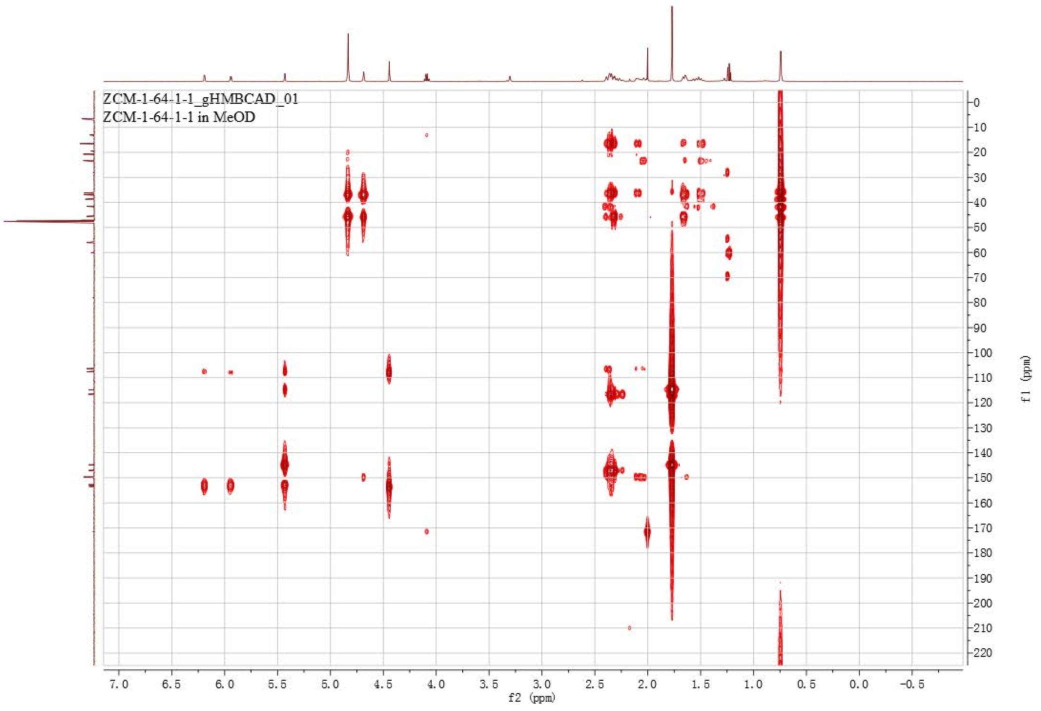

Figure S4.6. HR-APCI-MS spectrum of compound **4**

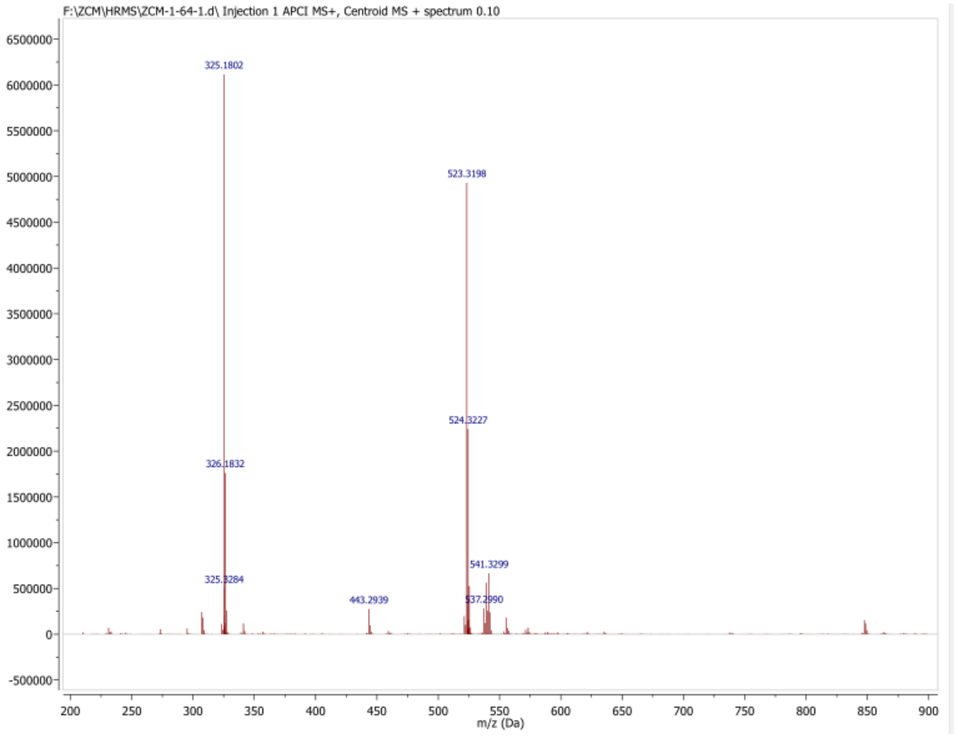

Figure S4.7. IR spectrum of compound **4**

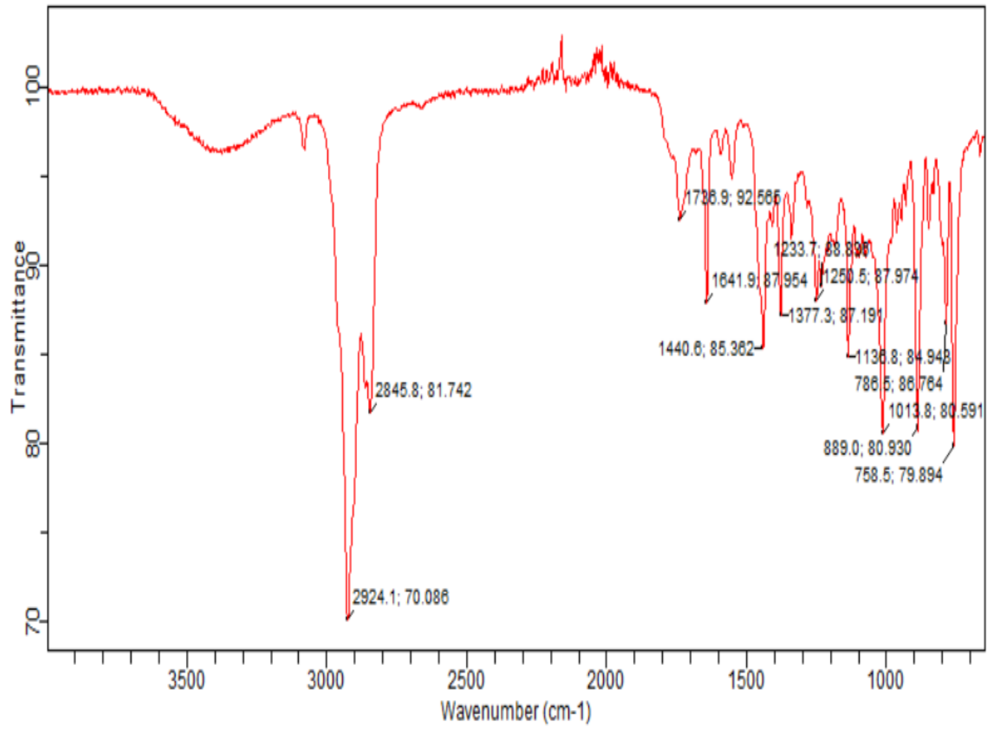

Figure S4.8. UV spectrum of compound **4**

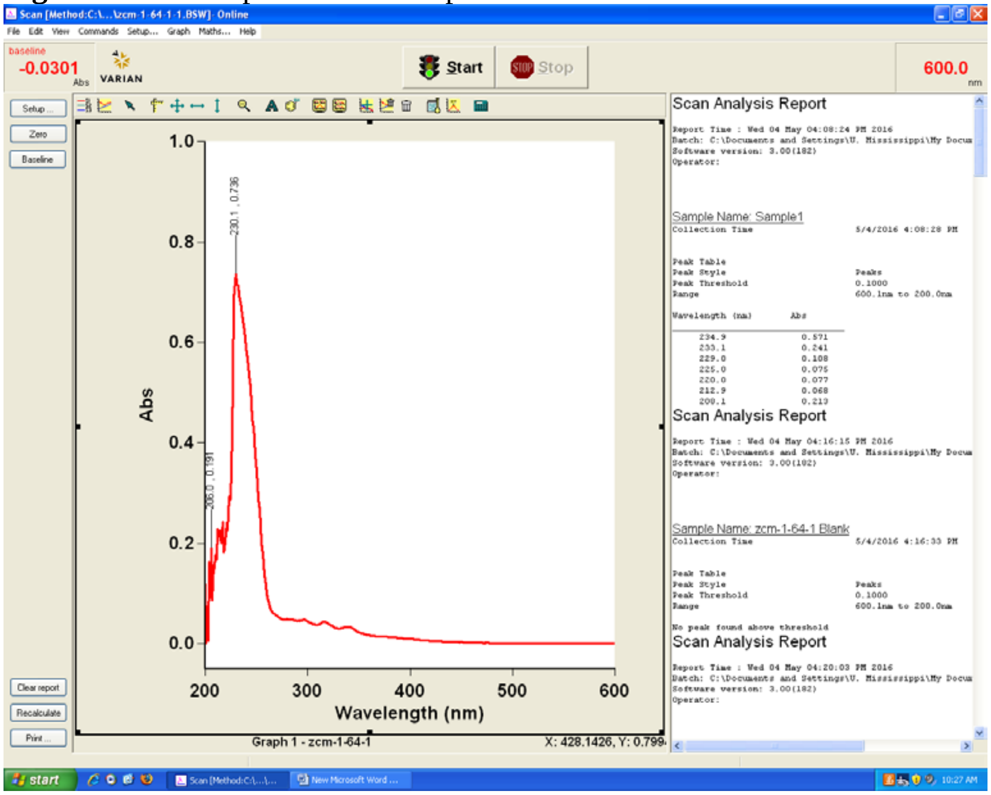

Figure S5.1. ^{1}H NMR spectral of compound **5**

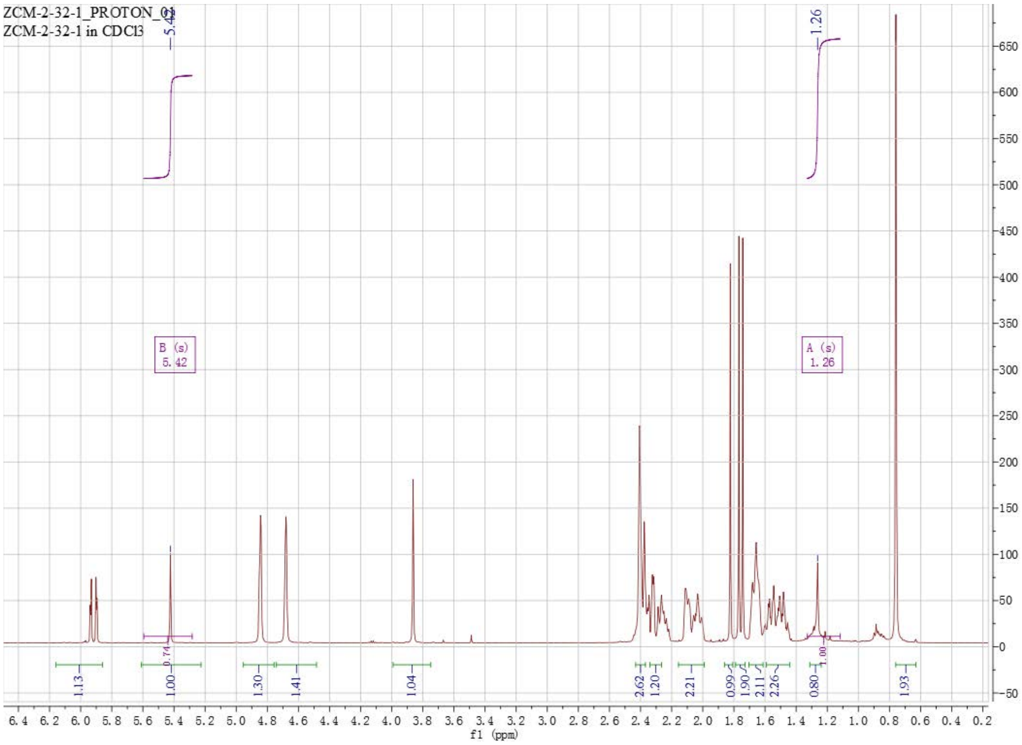

Figure S5.2. ^{13}C-NMR spectral of compound **5**

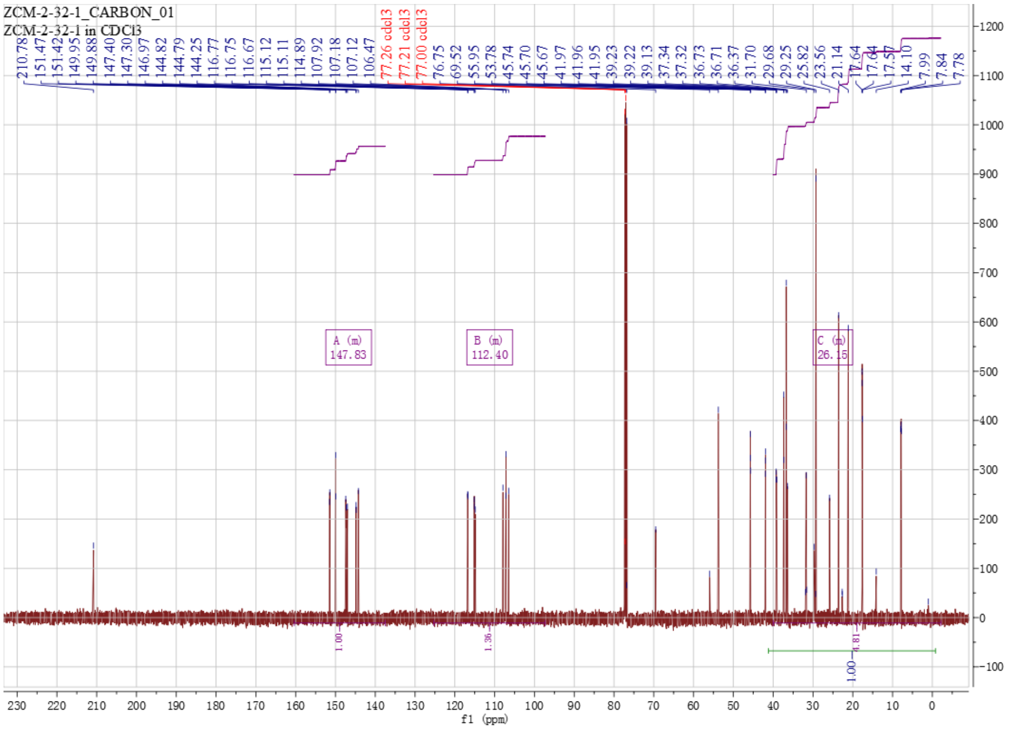

Figure S5.3. HSQC spectrum of compound **5**

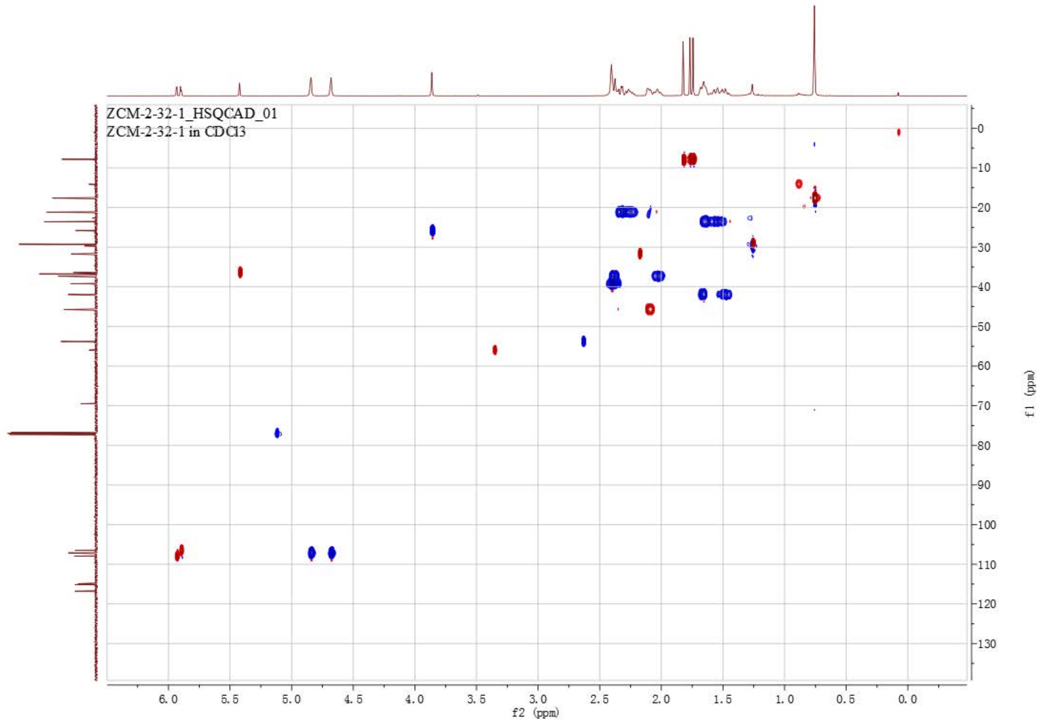

Figure S5.4. COSY spectrum of compound **5**

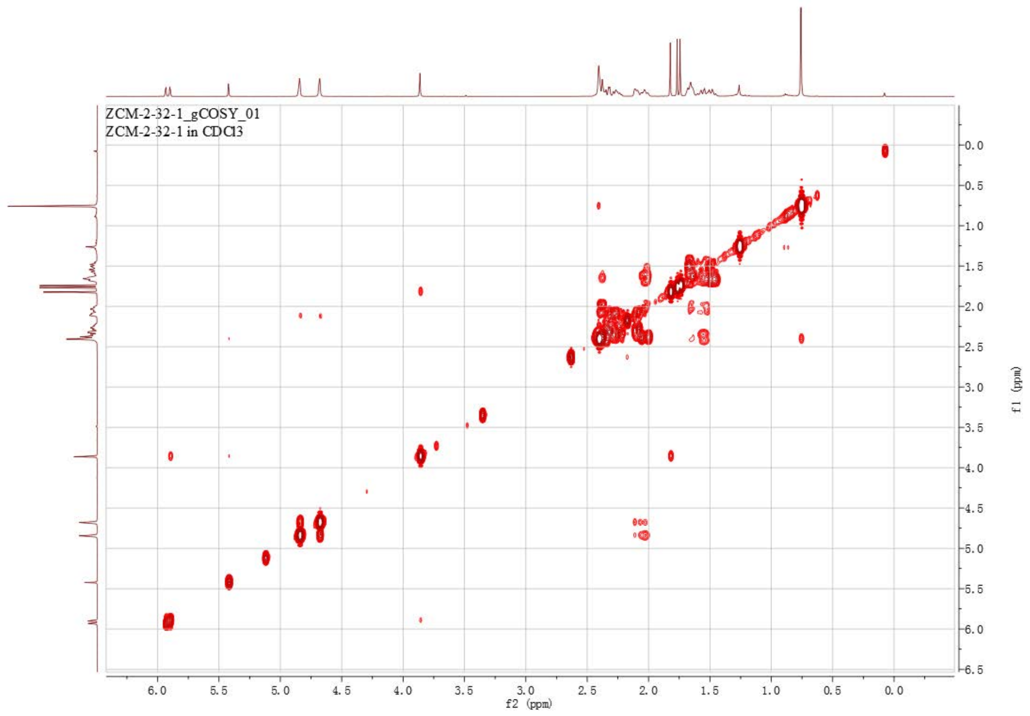

Figure S5.5. HMBC spectrum of compound **5**

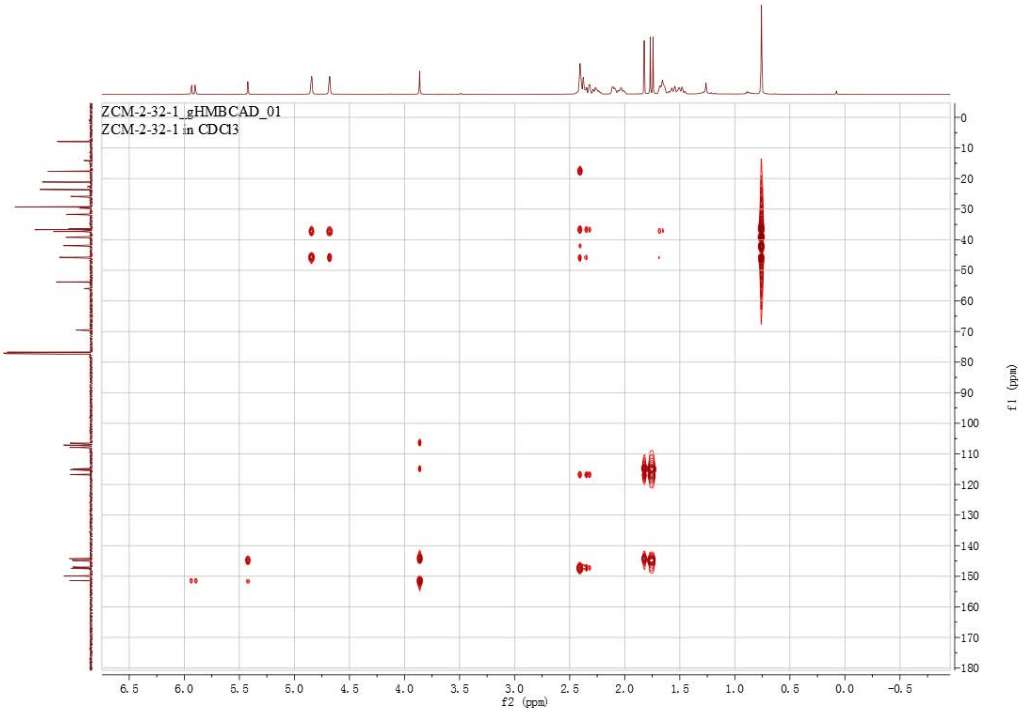

Figure S5.6. HR-APCI-MS spectrum of compound **5**

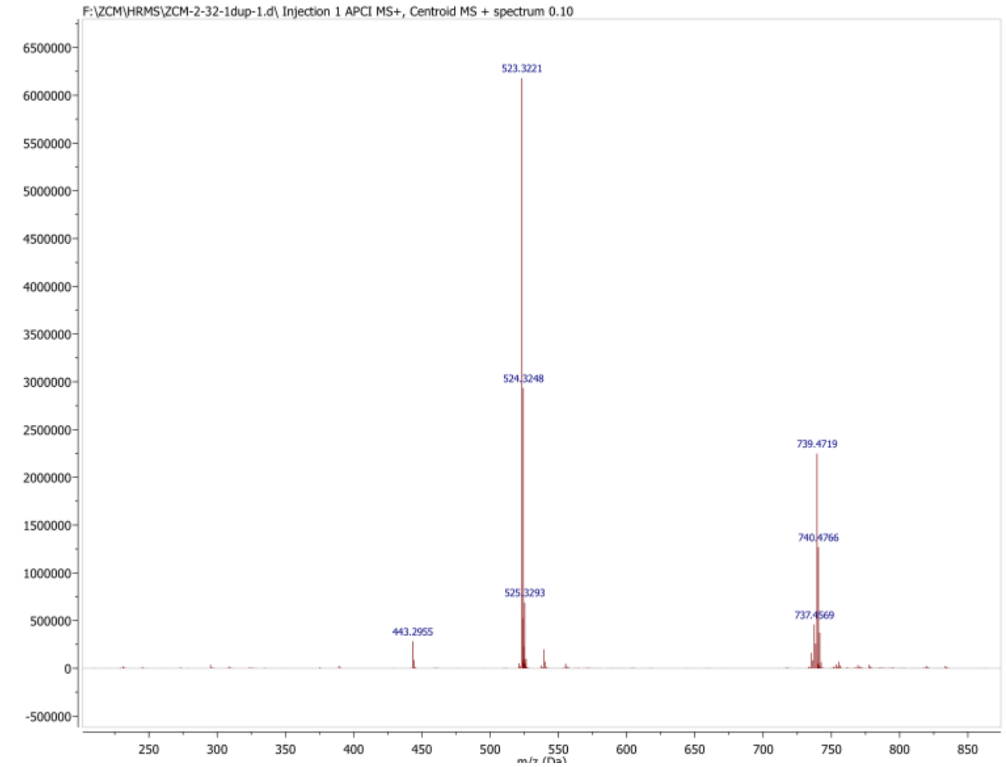

Figure S5.7. IR spectrum of compound **5**

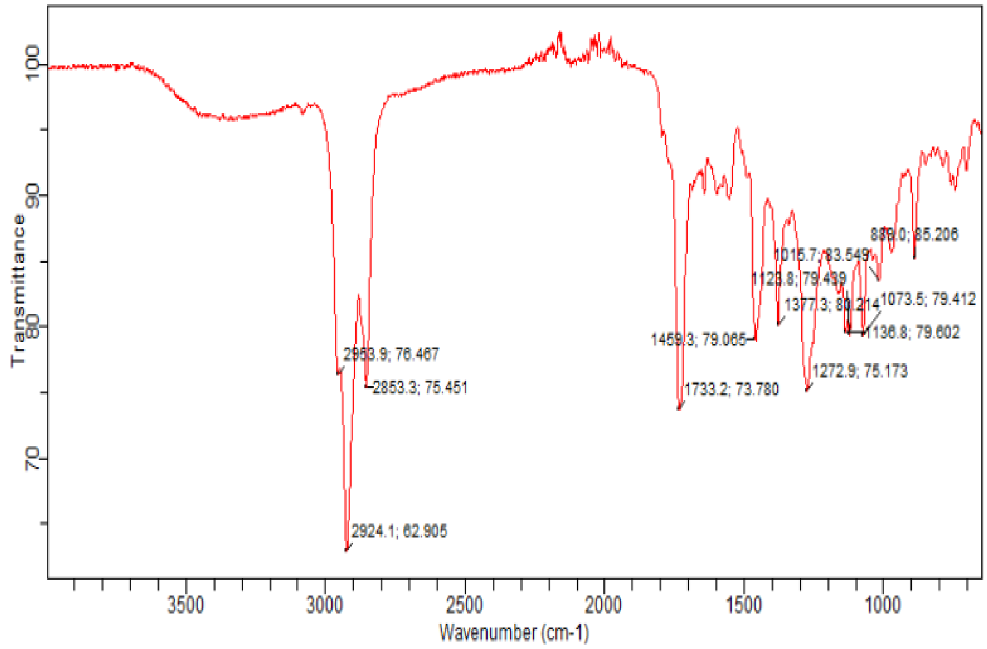

Figure S5.8. UV spectrum of compound **5**

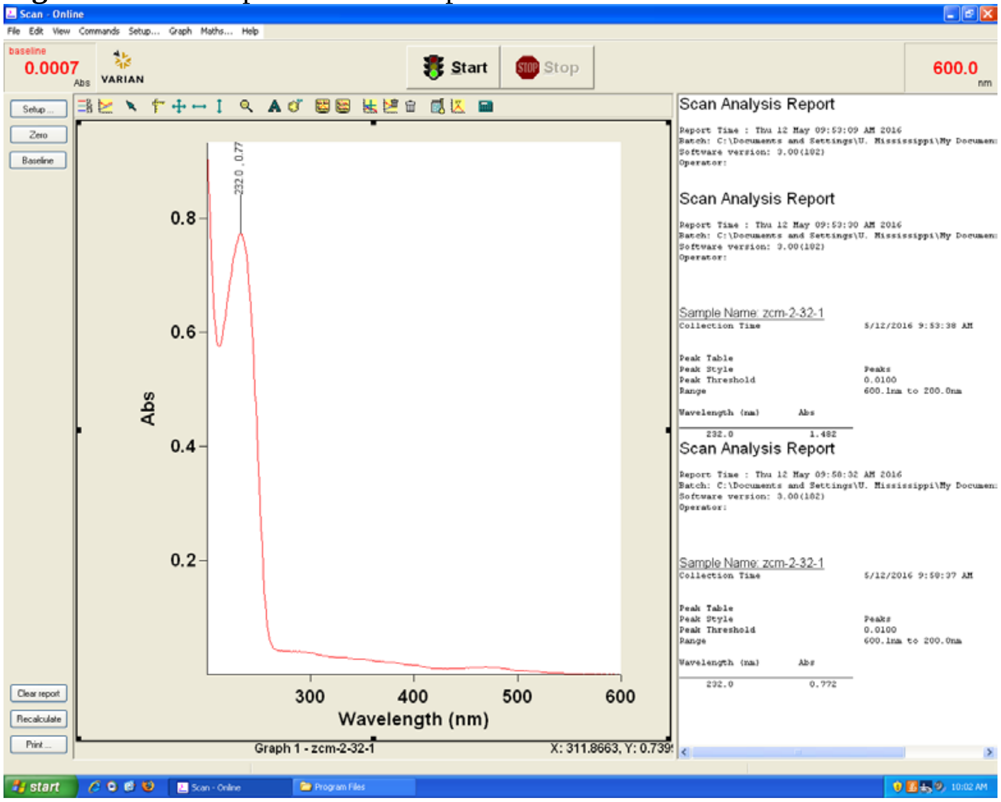

Supplement: Supplementary file 1 [file molecules-25-05904-s001.pdf]
